# *Ex vivo* enhancement of CD8+ T cell activity using functionalized hydrogel encapsulating tonsil-derived lymphatic endothelial cells

**DOI:** 10.7150/thno.100079

**Published:** 2025-01-01

**Authors:** Heesun Hong, Chan Hum Park, Ji Seung Lee, Kyunghee Kim, Sudarshini Nath, Moon Sik Oh, Sol Kim, Chul Hee Lee, Ki Hyun Kim, Woo Hee Choi, Kyu Young Choi, Hae Sang Park, Ok Joo Lee, In-Sun Hong, Soon Hee Kim

**Affiliations:** 1 Nano-Bio Regenerative Medical Institute, College of Medicine, Hallym University, Chuncheon 24252, Republic of Korea.; 2 Departments of Otorhinolaryngology-Head and Neck Surgery, Chuncheon Sacred Heart Hos-pital, School of Medicine, Hallym University, Chuncheon 24252, Republic of Korea.; 3 R&D Institute, ORGANOIDSCIENCES Ltd., Seongnam 13488, Republic of Korea.; 4 Department of Otorhinolaryngology-Head and Neck Surgery, Hallym University College of Medicine, Kangnam Sacred Heart Hospital, Seoul 07441, Republic of Korea.; 5 Department of Molecular Medicine, School of Medicine, Gachon University, Incheon 21565, Republic of Korea.

**Keywords:** Gelatin-hyaluronic acid based hydrogel, Tonsil derived lymphatic endothelial cells, Tonsil derived CD8^+^ T cells, T cell activation, Cancer immunology

## Abstract

**Rationale:** This study investigates a method for programming immune cells using a biomaterial-based system, providing an alternative to traditional *ex vivo* cell manipulation techniques. It addresses the limitations of engineered adoptive T cell therapies, such as T cell exhaustion, by introducing a gelatin-hyaluronic acid (GH-GMA) hydrogel system.

**Methods:** We characterized tonsil mesenchymal stem cells (TMSCs), lymphatic endothelial cells (T-LECs), stimulated T-CD8^+^ T cells (STCs), and GH-GMA biomaterials. The 10% 5:1 GH-GMA hydrogel, loaded with anti-CD28, cytokines interleukin-2 (IL-2) and vascular endothelial growth factor C (VEGF-C), forms a functional hydrogel capable of releasing these immune-stimulating factors. T-LEC spheroids, derived from tonsil mesenchymal stem cells (TMSCs), were encapsulated within the hydrogel to act as antigen-presenting cells for T cells.

**Results:** Co-encapsulation of STCs and T-LEC spheroids in the functional hydrogel resulted in significant expansion and enrichment of STCs during cultivation. Moreover, when cancer cells were co-encapsulated with STCs and T-LECs, there was increased migration of STCs towards the cancer cells and elevated expression of PD-L1 on the cancer cells.

**Conclusions:** These findings suggest that the GH-GMA hydrogel, combined with anti-CD28, IL-2, VEGF-C, and T-LEC spheroids, enhances T cell activity, presenting a promising platform for cancer immunotherapies and modulation of the suppressive tumor microenvironment.

## Introduction

T cells possess the capacity to migrate to and accumulate at sites of infection or disease, exhibiting a high specificity and sensitivity in destroying infected or mutated cells. However, dysfunctions may occur due to genetic or disease-related factors, compromising their effectiveness in defending against infections and malignancies 1. Adoptive immunotherapy with *ex vivo*-cultured T cells has been successful in treating various diseases, particularly viral infections and cancer 2. However, challenges like T cell exhaustion and hyporesponsiveness limit the full potential of these therapies and integrating biomaterials with engineered T cells aims to enhance therapeutic efficacy 3, 4.

T cell-extracellular matrix (ECM) interactions play a co-stimulatory role in T cell activation 5, 6. Members of the integrin family of adhesion molecules modulate the contact of T lymphocytes with other cells and ECM molecules, such as fibronectin, collagen, and laminin. In this context, biomaterials are pivotal in T cell activation for immunotherapy, as they have the capability to boost T cell activation, influencing their persistence, phenotype, and function. Therefore, biomaterials consisting ECM components has been used for T cell activation 7-9. Specifically, designing biomaterial platforms for T cell expansion enables precise control over the dosing, timing, and localization of adoptive T cells in the body. This control is critical for maintaining strong antitumor immune responses, optimizing therapeutic efficacy, and minimizing risks associated with off-target toxicities, T cell exhaustion, or immune escape 1, 10, 11.

Among biomaterials, hyaluronic acid (HA) is a non-sulfated glycosaminoglycan (GAG) present in the extracellular tissue of various parts of the body. HA is a versatile material for bioengineering and biomaterials due to its ease of physical and chemical modification, as well as its potential as an immune system modulator 12. Its role in the ECM is primarily focused on cell delivery systems, facilitating the transportation of therapeutic cells to the host's damaged site and the delivery of biologically competent cells, thus preventing unintended attacks by the host's immune cells 13. Particularly, the utilization of HA-based hydrogels presents a promising strategy for modulating the morphogenesis of lymphatic vessels in a biomimetic environment 14, 15.

Lymphatic vessels are essential for immune cell trafficking, facilitating the transport of immune cells, hormones, and maintaining fluid balance 16. Within lymph nodes, specialized lymphatic endothelial cells (LECs) interact with innate and adaptive immune cells, producing chemokines to recruit immune cells, including CD8^+^ T cells 17. Endothelial cells play a crucial role in recruiting and activating T-cells as part of the regulation of the immune system 18.

In this study, we hypothesized that the HA based hydrogel and the cytokines loaded within it would influence the lymphatic vessel formation of LECs, ultimately affecting T cell activation. In order to promote/facilitate CD8^+^ T cell activation and proliferation, we developed a functional hydrogel system based on photo-crosslinkable gelatin-hyaluronic acid (GH-GMA). The hydrogel was loaded with interleukin-2 (IL-2) and vascular endothelial growth factor C (VEGF-C), along with LECs spheroids. Notably, tonsillar tissue served as the source for both LECs and T cells in our study. Tonsils, crucial lymphoid organs, consist of epithelium from the endoderm and lymphoid tissue from the mesoderm, housing B and T follicular helper (Tfh) cells 19, 20. Tonsil tissues can be obtained from waste tissues resulting from tonsillectomies, are an attractive source for clinical applications. Also, TMSCs are promising candidates for regenerative medicine due to their self-renewal, high proliferation, tissue differentiation, immunomodulatory properties, and low immunogenicity 21-24.

First, we characterized TMSCs, LECs differentiated from TMSCs (T-LECs), and tonsil derived CD8^+^ T cells (T-CD8^+^ T cells), stimulated T-CD8^+^ T cells (STCs) and GH-GMA as a biomaterial. Next, we co-encapsulated STCs in the GH-GMA hydrogel loaded with IL-2 and VEGF-C, which also encapsulated T-LECs, and explored the interaction of STCs with T-LECs within it. Additionally, we examined how the functional hydrogel with T-LECs and T cells regulates the immune response in the tumor microenvironment (TME) by using *ex vivo* organotypic cultures with three solid tumor cell lines and analyzing the related immune checkpoints. Fostering T cell activation in a biomimetic and mild environment, we can expect that our system enhances the activity of CD8^+^ T cells, which could serve as a platform for various cancer immunotherapies or for the modulation of a suppressive TME.

## Methods

### Preparation and characterization of GH-GMA hydrogel

Gelatin (from porcine skin, 90-100 kD, gel strength 300, Type A, Sigma-Aldrich, St. Louis, MO, USA) and hyaluronic acid (HA, 15 kDa, Shiseido, Japan) modified with glycidyl methacrylate (GMA) (Gel-GMA and HA-GMA) were synthesized as described in the Supplementary data. Gel-GMA and HA-GMA solutions were mixed to produce the final GH-GMA. In brief, dialyzed Gel-GMA and HA-GMA solution were mixed with 3:1, 5:1, and 7:1 ratio (**Table 1**), and stirred for 2 h at room temperature (RT). Each mixture of GH-GMA solution was frozen at -80 °C for at least 24 h and freeze-dried for 7 days. Freeze-dried GH-GMA materials were stored at 4 °C for further analysis. Gel-GMA and HA-GMA were prepared as a control. GH-GMA were characterized by ^1^H NMR, Fourier Transform-Infrared Spectroscopy (FT-IR), X-ray diffraction (XRD), Scanning Electron Microscopy (SEM), and a rheometer (Supplementary data).

Each freeze-dried 5% HA-GMA, 10% Gel-GMA, 10% of 3:1, 5:1, and 7:1 GH-GMA was prepared in 1) phosphate-buffered saline (PBS) (Corning, NY., USA) containing 0.2% (w/v) of lithium phenyl-2,4,6 tri-methylbenzoylphosphinate (LAP) (Tokyo chemical industry, Tokyo, Japan) for degradation and swelling assay, 2) serum free RPMI1640 (Corning) media containing 0.2% (w/v) of LAP with 100 ng/ml of VEGF-C and 30 ng/ml of IL-2 for *in vitro* cytokine release assay, respectively. Pre-hydrogel was photo-polymerized with exposure to UV lamp at 365 nm with an intensity of 3 mW/cm^2^ at RT for 3 s. The degradation, swelling, and cytokine release assay were described in the Supplementary data. In the manuscript, we will refer to (pre-)hydrogels composed of media, LAP, IL-2, and VEGF-C as 'functional (pre-)hydrogels' for convenience.

### Primary culture of tonsil-derived mesenchymal stem cells (TMSCs)

Tonsils were obtained from children (ages 5-10) who underwent tonsillectomy in the period December 2018-March 2019 at Chuncheon Sacred Heart Hospital, Korea with patient consent from the legal guardians of all patients. This study was approved by institutional review board of Hallym University Medical Center (IRB No. 2017-87). For the isolation of tonsil cells, enzymatic digestion with collagenase type I and DNase I and density gradient methods were used (**Figure S1**). In details, tonsils were extensively washed twice with PBS containing 1% of penicillin-streptomycin (PS) (Gibco, Carlsbad, CA., USA), de-epithelialized by cutting with surgical scissor and enzymatically digested with 250 U/mL of collagenase type I (Invitrogen, Carlsbad, CA., USA) and 20 μg/ml of DNase I (Sigma-Aldrich, St. Louis, MO, USA) in Dulbecco's Modified Eagle's Medium (DMEM) (Corning) for 30 min at 37 °C in shaking incubator at 300 rpm. The cells were washed twice with DMEM supplemented with 30% Fetal bovine serum (FBS) (Corning) and 1% PS. Tonsil derived total cells were collected and then re-suspended in DMEM containing 20% FBS and 1% PS after filtration through cell strainer (100 µm, Falcon, NY., USA). The cell suspension was centrifuged for 10 min at 1500 rpm at 24 °C, and washed twice with DMEM supplemented with 20% FBS and 1% PS. Cell pellet was re-suspended in DMEM supplemented with 10% FBS and 1% PS. Then, it was slowly overlayed onto Ficoll-Paque (Histopaque-1077g/ml, Sigma-Aldrich), and centrifuged for 30 min at 2000 rpm at 24 °C. Mononuclear cells in white layer were collected carefully, and re-suspended in DMEM supplemented with 10% FBS and 1% PS. Density gradient centrifugation was processed for 3 times with 1500 rpm, 1300 rpm, and 1000 rpm for 10 min in order. Finally, cell pellet was re-suspended in DMEM supplemented with 10% FBS and 1% PS, and then plated at a density of 2 x 10^5^/cm^2^ of tonsil mononuclear cells in T-175 tissue culture flasks (Corning). The flasks were placed in a 5% CO_2_ incubator maintained at 37 °C. After 72 h, non-adherent cells were subsequently collected for purification of T cell population for further study. The plated TMSCs were cultured in DMEM supplemented with 10% FBS and 1% PS, with half-media changes performed every 3-4 days. When the confluency of TMSCs reached 80%, they were sub-cultured using 0.25% Trypsin-EDTA solution (Gibco). Morphological changes were detected by inverted microscopic observation.

### Generation of tonsil derived lymphatic endothelial cells (T-LECs) & spheroids

Cultured TMSCs at passage 3 were used for generation of T-LECs. Briefly, culture plates were coated with 10 μg/ml of fibronectin (FN, Corning) for 45 min in CO_2_ incubator at 37 °C, and cultured T-LECs were prepared concentration at 3 x 10^4^/cm^2^ in EGM-2MV medium (EGM^TM^-2 MV BulletKit^TM^, Lonza, Houston, TX., USA) supplemented with 100 ng/ml of VEGF-C (Biolegend, San Diego, CA., USA). Harvested T-LECs were plated on FN and cultured for 21 days, with sub-culturing at a 1:3 dilution when the confluency reached 80% using 0.25% Trypsin-EDTA solution. Induced T-LECs at passage 3-5 were used for further experiments. The changes in cell morphologies were analyzed during induced differentiation of TMSCs to T-LECs at passage 3 using an inverted phase-contrast microscope (Nikon, Japan).

A spheroid culture system was used to provide a 3D cell culture condition for T-LEC spheroid formation. At day 21 of induction, the T-LECs were harvested, and then re-suspended in EGM-2MV medium supplemented with 500 ng/ml of VEGF-C. Subsequently, 5 x 10^3^ cells/200 μl of T-LECs were plated in each well of an ultra-low attachment 96-well plate (96-well Clear Round Bottom Ultra-Low Attachment Microplate, Corning). Cells were cultured for 5-7 days in a CO_2_ incubator at 37 °C to form spheroid. During culture period, 150 μl of media was removed and an equal volume of fresh complete media was added to each well of the culture plate. Some T-LEC spheroids were labeled with CellTracker-conjugated-red (Ex577, Em602, CellTracker^TM^ Red CMTPX, ThermoFisher, Waltham, MA., USA) according to the manufacturer's instruction.

### Tube formation assay using T-LECs

The tube formation assay was conducted to functionally evaluate the 3D network structure formation of induced T-LECs using Matrigel-based assay. In detail, 50 μl of growth factor-reduced Matrigel^TM^ (Corning) was added to each well of a 96-flat-bottomed well plate (Falcon). After allowing the plate to polymerize for 30 min in a CO_2_ incubator maintained at 37 °C, induced T-LECs at a concentration of 1 x 10^4^ cells/well were harvested and suspended in EGM-2MV medium supplemented with 500 ng/ml of VEGF-C. Next, 100 μl of the T-LECs suspension was seeded into the 96-flat-bottomed well plate containing Matrigel^TM^ and incubated overnight in the CO_2_ incubator at 37 °C. Non-differentiated TMSCs were used as a control group.

### Viability, proliferation, & sprouting of T-LEC spheroids encapsulated in hydrogels

T-LEC spheroids were encapsulated in 10% 3:1, 5:1, and 7:1 GH-GMA pre-hydrogels containing of 0.2% (w/v) LAP in EGM-2MV supplemented with 100 ng/ml of VEGF-C, and photo-polymerized with UV exposure at 365 nm for 3 s. Then, T-LEC spheroids encapsulated in hydrogels were incubated at 37 °C in 5% CO_2_ incubator. Live & Dead assay, proliferation assay using CCK-8 and sprouting assay were performed as follows.

#### Live & Dead assay

The detection of the viability of encapsulated T-LEC spheroids in hydrogel during culture periods was performed using live & dead assay. Briefly, cultured T-LEC spheroid at day 3, and day 7 were collected, and washed with DPBS, then incubated with 2 μM Calcein-AM and 4 μM ethidium homodimer-1 solution for 30 min in 5% CO_2_ incubator maintained 37 °C. After incubating, T-LEC spheroids were washed with DPBS, and observed and imaged using confocal microscope (K1-fluo, Nanoscope Systems, Korea).

#### Proliferation assay

Cell Counting Kit-8 assay (CCK-8, EnzoBiochem, New York, NY, USA) was carried out proliferation assay according to the instruction from manufacturer. The culture supernatants were collected during the cultivation of T-LEC spheroids in hydrogel on days 3 and 7. Specifically, 100 μl of culture supernatant was collected per well of a 96-well plate (SPL, Korea). 10 μl of CCK-8 assay reagent was added per well, after 3 h incubation for at 37 °C in a CO_2_ incubator, the optical absorbance was measured using a microplate reader (BioTek, Winooski, VT, USA) at 450 nm. Serum free media was used as a control, experiments were performed at least three times.

#### Sprouting assay

Lymphatic vessel formation (sprouting assay) was performed in situ within the pre-hydrogel. In detail, 10% of 3:1, 5:1, and 7:1 of GH-GMA containing 0.2% (w/v) of LAP was prepared with EGM2-MV culture media supplemented 100 ng/mL of VEGF-C. One T-LEC spheroid was encapsulated in various concentration of GH-GMA hydrogel. Pre-hydrogels were photopolymerized with exposure to UV lamp at 365 nm with an intensity of 3mW/cm^2^ at RT for 3 s, and incubated in 5% CO_2_ incubator at 37. After 24 h, images were obtained with an inverted microscope (Carl Zeiss, Jena, Germany).

### Purification of tonsil derived CD8^+^ T cells (T-CD8^+^ T cells)

T-CD8^+^ T cells were obtained from the non-adherent cells within tonsil cells. In brief, isolated tonsil cells were cultured to induce TMSCs with adherent tonsil cells. Meanwhile, the tonsil-derived non-adherent cells (TMCs) were collected for the purification of CD8^+^ T cells. The cells were suspended in RPMI1640 supplemented with 10% FBS and 1% PS and then incubated for 2 days in a CO_2_ incubator at 37 °C. After cultivation, the cells were washed twice with DPBS containing 2% FBS and then re-suspended in FACS buffer. As a positive control, human peripheral blood mononuclear cells (PBMCs) were purchased from PromoCell (Heidelberg, Germany). PBMCs were thawed, and washed with RPMI1640 supplemented with 10% FBS and 1% PS and then incubated for 2 days in a CO_2_ incubator at 37 °C. For the purification of CD8^+^ T cell, CD8 APC monoclonal antibody and isotype APC Mouse IgG_1_, κ APC (both from Biolegend) as a control were used for sorting by flow cytometer. In detail, cells were stained with antibody for 30 min at 4 °C in the dark, and washed with DPBS containing 2% FBS. The stained TMCs and PBMCs (1 x 10^7^/ml) were sorted using an 85 μm nozzle, and the total threshold count was 6,627,252 by FACSAria II (BD biosciences, San Jose, CA., USA). The analysis of purity and yield was conducted using FACSDiva software (version 6.1.3, BD Biosciences) with the CD8-positive portion identified in quadrant gating.

### Stimulation/activation of T-CD8^+^ T cells (STCs)

To activate the tonsil or PBMC derived CD8^+^ T cell, 5.0 μg/ml of anti-CD3 (Biolegend) was plated into each well of a 6-well plate (SPL, Korea). The plate was then incubated in a CO_2_ incubator at 37 °C. After 3 h, the anti-CD3 solution was removed, and the sorted tonsil or PBMC derived CD8^+^ T cell (at a concentration of 2 x 10^5^ cells/ml) were re-suspended in RPMI1640 supplemented with 10% FBS and 1% PS. To this medium, 1 μg/ml of anti-CD28 and 5 ng/ml of IL-2 (both from Biolegend) were added. Subsequently, the cell suspension was re-plated onto an anti-CD3 coated 6-well plate and incubated for 3 days (Day 0) in CO_2_ incubator at 37 °C. After the 3 days of incubation (Day 3), 1 ml of fresh culture medium consists with RPMI1640 supplemented with 10% FBS, 1% PS, and 5 ng/ml of IL-2 (expansion medium) was added to each well. Another 3 days later (Day 6), the CD8^+^ T cells were transferred to an anti-CD3 coated 60 mm dish (SPL, Korea) with the expansion medium, and 2 days later (Day 8), 2 ml of fresh expansion medium was added to each well. At Day 10, enriched tonsil or PBMC derived CD8^+^ T cells were used for further experiments. Some of CD8^+^ T cells were labeled with CellTracker-conjugated-Green (Ex492/Em517, CellTracker^TM^ Green CMFDA, ThermoFisher) according to manufacturer's instruction. Tonsil or PBMC derived CD8^+^ T cells were counted by using automatic cell counter. Viability, immunophenotyping, and intracellular protein detection were performed using a flow cytometer. Additionally, the secretion of IL-2 and IFN-γ was evaluated at days 3 and 10 during the expansion procedure.

### T-LEC spheroid with STCs co-encapsulated in hydrogels

To determine the interaction of STCs with T-LEC spheroids, T-LEC spheroids and STCs (ratio 1:10, T-LECs 3 x 10^4^ cells *vs* 3 x 10^5^ STCs) were mixed with the functional pre-hydrogel (10% 5:1 GH-GMA containing of 0.2% (w/v) LAP in RPMI1640 supplemented with 30 ng/ml of VEGF-C, 10 ng/ml of IL-2, and 1 μg/ml of anti-CD28). Then, it was photo-polymerized with UV exposure at 365 nm for 3 s. After 1 and 3 days incubation with RPMI1640 and EGM-2MV (ratio 1:1) supplemented with 10% FBS and 1% PS, ELISA and immunofluorescent staining was performed (refer to the “IF staining” and “ELISA” section).

### Generation of cancer spheroids

Three human malignancy cancer cell lines were used in this study: AGS, a gastric cancer cell line; A549, lung cancer cell line; and MDA-MB-231, a metastatic breast cancer cell line. These cell lines were kindly gifted from Dr. Lee, Hai-Chon at Scripps Korea Antibody Institute (Chuncheon, Korea). All cancer cell lines were cultured in a CO_2_ incubator maintained at 37 °C. AGS cells were cultured in F-12 nutrient mixture (Gibco), A549 cells in DMEM, and MDA-MB-231 cells in RPMI1640 supplemented with 10% FBS and 1% PS. The cells were sub-cultured every 3-4 days using 0.25% Trypsin/EDTA solution when the confluency reached 70%. For tracking purposes, each cancer cell line was labeled with CellTracker-conjugated-violet (Ex 415, Em 516, CellTracker^TM^ Violet BMQC dye, ThermoFisher) according to the manufacturer's instruction. The labeled cancer cells were plated to initiate spheroid formation. For each cancer cell line, 5 x 10^3^ cells/200 μl were collected and plated in an appropriate culture medium into each well of an ultra-low attachment round-bottomed 96-well microplate. The plate was then incubated in a CO_2_ incubator maintained at 37 °C for 7 days. During the culture period, 150 μl of media was removed, and an equal volume of fresh media was added to each well every 3 days.

### STCs migration assay using transwell

The migration assay of STCs was performed using transwell cell culture inserts (BD Biosciences) with a diameter of 6.5 mm and 4-µm pore filters. In the lower layer, 2 x 10^4^/ml of AGS, A549, and MDA-MB-231 cancer cells were plated in appropriate culture medium. After 3 days of incubation, the cultured medium was removed. Meanwhile, in the upper layer, T-LEC (3 x 10^4^) spheroids and STCs (3 x 10^5^/ml) were plated in culture medium containing RPMI1640 and EGM-2MV (ratio 1:1) supplemented with 30 ng/ml of VEGF-C and 10 ng/ml of IL-2. Following incubation for 24 h, for the detection of T cell migration, unmigrated cells from the upper chamber were aspirated and wiped off with a cotton swab, and incubated with 4% PFA for 10 min. Subsequently, the chamber was washed and stained with 1 μg/ml of DAPI for 1 min at RT, and washed with PBS. Stained cells were observed and visualized under confocal laser scanning microscope (K1-fluo, Nanoscope Systems, Korea). Also, real-time quantitative reverse-transcript-polymerase chain reaction (qRT-PCR) was performed (refer to the “qRT-PCR” section).

### *Ex vivo* reconstituted 3D organotypic culture with cancer spheroids

To reconstitute a 3D cancer organotypic culture using various cancer spheroids, a 10% 5:1 GH-GMA pre-hydrogel solution was prepared with the appropriate culture medium; F-12 nutrient mixture was used for AGS, DMEM for A549, and RPMI1640 for MDA-MB-231. All culture medium was supplemented with 10% FBS, 1% PS, and 0.2% (w/v) LAP. Each labeled cancer spheroid was mixed with 10 μl of 10% 5:1 GH-GMA pre-hydrogel solution in a 35 mm culture dish (Corning). Meanwhile, T-LEC spheroids and STCs were mixed with the functional pre-hydrogel (10 % of 5:1 GH-GMA pre-hydrogel prepared in RPMI1640 supplemented with 30 ng/ml VEGF-C, and 10 ng/ml of IL-2, and 1 μg/ml of anti-CD28) with 5% FBS and 1% PS. Then, 20 μl of this cell suspension were poured onto the above cancer spheroid laden pre-hydrogel solution, and photo-polymerized with UV exposure at 365 nm for 3 s. Next, this 3D cancer organotypic culture system was incubated for 3 days in CO_2_ incubator maintained at 37ºC. The culture medium consisted of EGM-2MV and the appropriate culture medium for each cancer type supplemented with 5% FBS and 1% PS. The cell combination ratio of cancer spheroids : T-LEC spheroids : STCs was 1:1:10, with a cell dose of 3 x 10^4^ : 3 x 10^4^ : 3 x 10^5^.

### Immune checkpoint PD-L1

For the detection of PD-L1 on various cancer cell lines, a transwell culture procedure was used with 6.5 mm in diameter and 8-µm pore filters. In the lower layer, three type of cancer cells (2 x 10^4^/ml) were plated in appropriate culture medium. After 3 days of incubation, the cultured medium was removed. Meanwhile, in the upper chamber of the transwell plate, T-LEC (3 x 10^4^) spheroids and STCs (3 x 10^5^/ml) encapsulated in 30 μl of functional pre-hydrogel (10% 5:1 GH-GMA hydrogel, which was prepared using RPMI1640 and EGM-2MV (in a 1:1 ratio) supplemented with 30 ng/ml of VEGF-C and 10 ng/ml of IL-2) were placed. This chamber was inserted to lower chamber and incubated in a CO_2_ incubator maintained at 37 °C for 3 days. After cultivation, the cancer cells in the lower layer were harvested using 0.25% Trypsin/EDTA, and then washed with DPBS. Subsequently, three type of cancer cell lines were stained with PD-L1 PE (Biolegend) at 4ºC for 30 min. Each cell suspension was re-suspended in 500 μl of FACS buffer for acquisition on flow cytometer using FACSCalibur (BD Biosciences) (refer to the “Flow cytometer” section).

### Real-Time Quantitative Reverse-Transcript-Polymerase Chain Reaction (qRT-PCR)

Total RNA was extracted from cultured TMSCs, induced adipogenesis, chondrogenesis, and osteogenesis from TMSCs, as well as induced T-LECs cells and spheroids, using Trizol Reagent (Invitrogen) with the chloroform-isopropyl alcohol purification method. Subsequently, the RNA pellets were washed with 75% ethyl alcohol and air-dried for 15 min at RT. After dissolving 20 μl in RNase-free DEPC water, the purity and concentration of total RNA were measured using a spectrophotometer (Molecular Devices, Sunnyvale, CA, USA). For the synthesis of cDNA, 1 μg of RNA was utilized with Maxime RT Premix (Oligo dT, Intron Biotechnology, Seoul, Korea) following the manufacturer's instructions. The primers used were as follows; 1) TMSC stemness genes: OCT-4, SOX-2, NANOG, and c-myc, 2) Tri-lineage differentiation specific genes: LPL, PPARG, FABP4 for adipogenesis; COL2A1, Aggrecan, SOX-9 for chondrogenesis; and OC, ALPL, BMP2 for osteogenesis. 3) T-LEC and spheroids genes: CD31, KDR (Flk1), VEGF-R3 (FLT4), Prox-1, LYVE-1, and podoplanin, 4) T cell migration related genes (CCR7, CXCR3, LFA-1, and ICAM-1), and 5) TME and apoptosis-related genes (perforin, granzyme, CXCR3&5, CCL19&21, MMP8&9). The PCR was performed using PCR pre-mix reagent (Maxime^TM^ PCR PreMix, Intron Biotechnology). PCR products were separated by electrophoresis on 1% (w/v) agarose gels (LE agarose, GenomicsOne, Seoul, Korea) using Loading dye (LoadingStar, DawinBio, Seoul, Korea), and the resulting bands were visualized under UV. For qRT-PCR, SYBR Green mixture (Applied Biosystems, Waltham, MA., USA) was employed, and the real-time PCR instrument (QuantStudio 3 Real-Time PCR Instrument, Applied Biosystems). The PCR protocol involved an initial denaturation for 10 min at 95 °C, followed by 40 cycles of denaturation at 95 °C for 30 s and annealing/extension at 60 °C for 45 s per cycle. The gene expression data was normalized with GAPDH (glyceraldehyde-3-phosphate dehydrogenase) as a reference gene using the 2-ΔΔCt method. All primers used in this study were presented in **Table S1**. To ensure the reproducibility of the results, all experiments were repeated at least three times.

### Flow cytometer

0.5-1 x 10^6^ cells from TMSCs, T-LECs, T cells, A549, AGS, and MDA-MB231 cancer cell lines were washed with PBS and stained with surface or intracellular monoclonal antibodies in 100 μl of FACS buffer (Biolegend). Cell viability was determined using 7-Aminoactinomycin D (7-AAD) solution (7-AAD viability staining solution, Biolegend) following the manufacturer's instructions. For intracellular staining, STCs were incubated with 100% cold methanol for membrane permeabilization, then washed twice with FACS buffer, and subsequently stained with IL-2 and IFN-γ. The following anti-human monoclonal antibodies were used in this study: 1) CD44 PE, CD73 APC, CD90 FITC, CD105PerCP-Cy5.5, and CD11b/CD19/CD34/CD45/HLA-DR PE (Human MSC Analysis Kit #562245, BD Biosciences) for TMSCs; 2) podoplanin APC, VEGF-R3 PE (both Biolegend), ALEXA FLUOR^®^ 488-conjugated Prox-1 (Bioss, Woburn, MA., USA), and ALEXA FLUOR^®^ 488-conjugated LYVE-1 (R&D Systems, Minneapolis, MN., USA) for T-LECs; 3) CD4 PE, CD8 APC, CD44 FITC, CD62L PE, CD69PE (Miltenyi Biotec, Westphalia, Germany), IL-2 PE, and IFN-γ FITC(Biolegend) for T cells sorting or STCs; 4) PD-L1 PE (Biolegend) for TME. The cell suspensions with each antibody were incubated for 30 min at 4 °C while avoiding exposure to light. Then, the cells were washed twice and finally re-suspended in 500 μl of FACS buffer for acquisition on a flow cytometer using the FACSCalibur and FACSCanto II System (BD Biosciences). A total of 10,000 cells were counted, and non-stained TMSCs and isotype control for each wavelength were used as controls. Data were analyzed using CellQuest Pro^TM^, FACSDiva^TM^ and Flowjo^TM^ (BD Biosciences). The results were presented as the percentage and fluorescent intensity of cells labeled for each monoclonal antibody. **Table S2-3** showed the list of monoclonal antibodies in this study.

### Immunofluorescent staining

For the immunofluorescent staining of T-LECs followed tube formation, podoplanin, LYVE-1, Prox-1, and VEGF-R3 as LECs specific marker were used, and induced T-LEC spheroids were stained with podoplanin only. Cultured T-LEC cells on Matrigel and induced T-LEC spheroids were fixed in 4% PFA for 30 min at RT, and washed three times using PBS, respectively. The fixed cells and spheroids were blocked with 1% bovine serum albumin (BSA, Merck, St. Louis, MO., USA) solution in PBS solution with 0.1% Tween-20 (PBST, Sigma-Aldrich) for 30 min at RT. The T-LECs were incubated with Alexa Fluor 594 conjugated anti-human podoplanin (1:100), Alexa Fluor 594 conjugated anti-human LYVE-1 (1:100), Alexa Fluor 488 conjugated anti-human Prox-1 (1:100), and Alexa Fluor 488 conjugated anti-human VEGF-R3 (1:100), and for T-LEC spheroids were incubated with Alexa Fluor 594 conjugated anti-human podoplanin (1:100) at 4 °C overnight. The Following day each cell was washed 3 times with PBS, and counterstained with 1 μg/ml of DAPI for 1 min at RT, and washed with PBS. Stained cells were observed and visualized under confocal laser scanning microscope (Carl Zeiss) using analysis program (ZEN, Carl Zeiss).

### Measurement of cytokine secretion by ELISA

For the detection of IL-2 (Abcam, Cambridge, UK), the manufacturer's instructions were followed. The samples were prepared during the cultivation of STCs with T-LEC spheroids or during the expansion of STCs. In detail, 50 μl of each sample and standard of IL-2 for STCs were added to each well on 96-well microplates. Subsequently, 50 μl of IL-2 antibody was added to each well, and the plate was incubated for 1 h at RT on a plate shaker at 300 rpm. After three washes, Horse Radish Peroxidase (HRP) conjugated TMB (3,3',5,5'-tetramethylbenzidine) substrate solutions were added for colorimetric development, and the plate was incubated on the plate shaker at 300 rpm. After 10 min, the stop solution was added to each well and shaken for 1 min on a plate shaker.

For IFN- γ detection, the Human IFN-gamma ELISA kit (Abcam) was used, and the procedure followed the manufacturer's instructions. Briefly, each sample from STCs expansion and the standard were added to each well in a 96-well microplate. Then, 1x biotinylated anti-IFN-γ was added to all wells, and the plate was incubated 2 h at RT. After the incubation, each well was washed three times, and 1 x streptavidin-HRP solution was added to all wells, followed by a 30-min incubation. Subsequently, the plate was incubated with Chromogen TMB substrate for 15 min, and the reaction was stopped by adding the stop solution into each well.

For all ELISA experiments of IL-2 and IFN-γ, the results were detected by optical density (O.D. value) at 450 nm using a spectrophotometer (BioTek instruments, Winooski, VT., USA).

### Statistics

Statistical analysis was performed using GraphPad Prism 5.0 software (Graphpad, San Diego, CA, USA) with one- or two-way ANOVA (analysis of variance) or unpaired, two-tailed Student's *t*-test. Results are presented as mean ± standard deviation (mean ± S.D.). Statistical significance was set as follows: *p* < 0.05 (*), *p* < 0.01 (**), and *p* < 0.001 (***); non-significant results are marked as 'ns'. All experiments in this study were performed at least triplicate for data reproducibility.

## Results and Discussion

### Characterization of functionalized biomimetic hydrogel

In this study, hyaluronic acid (HA) was chosen as a cellular carrier material to enhance the morphogenesis of lymphatic vessels from LECs. This promotes interaction between CD8^+^ T cells and LECs, ultimately enhancing the interaction with cancer cells. HA with high biocompatibility is widely used in bio-scaffolds and injectable hydrogels, showing positive results in *in vitro* and *in vivo* tests for tissue regeneration. However, unmodified HA has drawbacks like weak mechanical properties and quick *in vivo* degradation. These limitations can be addressed through chemical modification or crosslinking, enhancing mechanical strength, degradation control, viscosity, solubility, and biological properties 25, 26. Here, we synthesized methacrylated derivatized HA using glycidylmethacrylate (GMA) to improve mechanical properties of HA 27, and combined with gelatin to improve cell attachment and to control swelling and degradation ratio of HA 28, 29. Gelatin also was crosslinked because unmodified gelatin degrades above 30 °C and is mechanically weak and unstable 30.

**Figure 1:**
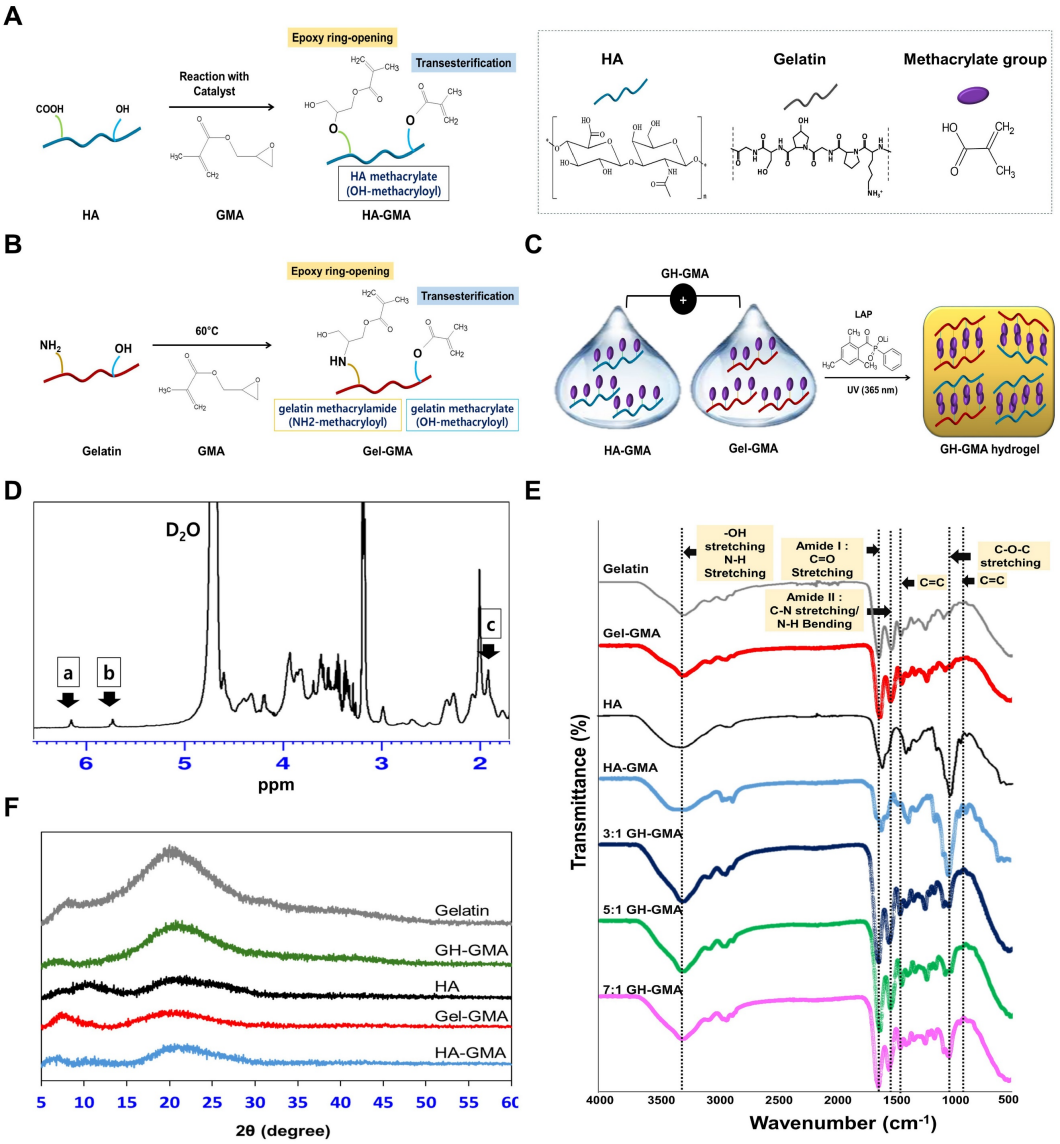
** Synthesis, preparation, and characterization of GH-GMA pre-hydrogel. (A)** Synthetic scheme of HA-GMA: Hyaluronic acid (HA) is modified with glycidyl methacrylate (GMA) through reaction with carboxyl or hydroxyl moieties on HA. **(B)** Synthetic scheme of Gel-GMA: Gelatin is conjugated with GMA, reacting with primary amine or hydroxyl groups on the gelatin. Both reactions proceed through transesterification or epoxy ring opening. **(C)** Schematic representation of GH-GMA hydrogel formation: Crosslinking between Gel-GMA and HA-GMA produces a hybrid GH-GMA hydrogel. UV irradiation with LAP generates free radicals, crosslinking liquid GH-GMA via vinyl polymerization. **(D)** ^1^H-NMR of GH-GMA pre-hydrogel. Peaks at δ = 6.2 ppm and δ = 5.7 ppm (a, b) correspond to the methacrylate group, while the peak at δ = 1.9 ppm (c) represents methyl protons.** (E)** FT-IR Spectroscopic analysis, and (**F**) X-ray diffraction (XRD) patterns (see **Figure S2** for detail pattern).

**Figure 1A-C** shows synthesis and preparation of hydrogels used in this study. We anticipated that the HA-GMA and Gel-GMA were synthesized by methacrylate substitution of the carboxylic acid groups, primary amines and hydroxyl group in gelatin and the carboxylic acid groups and primary hydroxyl group in HA, respectively, by ring opening or transesterification reaction 31-33. Upon UV irradiation, LAP initiates free radical generation, enabling vinyl polymerization and crosslinking of the liquid GH-GMA. Hydrogel stiffness, modulated by adjusting concentration, functionalization degree, UV exposure, and additives 34, is directly influenced by material concentration but requires precise balancing to support cell viability and function. In this study, we used 10% GH-GMA, with gelatin below 10% and HA (15 kDa) below 5% based on previous reports 35, 36. Previous findings suggest that 10% gelatin in GelMA enhances T-cell IL-2 release over 20% 37, and MSCs show better differentiation and proliferation below 10% GelMA 34. Low molecular weight HA (<35 kDa) is associated with promoting angiogenesis, with oligomers in the 6-20 kDa range activating antigen-presenting cells like dendritic cells, and slightly larger HA (20-450 kDa) reported to stimulate inflammatory cytokines 38.

**Figure 1D** presents the ^1^H NMR spectra of the GH-GMA pre-hydrogel. The introduction of glycidyl methacrylate is evident from the vinyl hydrogen signals at 5.7 ppm and 6.2 ppm, which indicate attachment to the gelatin or HA structure. Additionally, the methyl proton peak at 1.9 ppm confirms the presence of methacrylate groups. The peak at 2 ppm (signal for methyl group of N-acetylglucosamine) and complex splitting around 3.2 ppm and corresponds to the native structure of HA 39. In the gelatin portion, distinct peaks at 2.3 ppm, 2.7 ppm, and 2.9 ppm align with the characteristic signals for glutamic acid (or hydroxyproline), aspartic acid, and lysine residues, respectively, highlighting the native gelatin structure 40. GH-GMA pre-hydrogel was characterized using a Fourier-transform infrared spectroscopy (FT-IR) to confirm the presence of characteristic functional groups in composites (**Figure 1E**). HA shows a broad peak at 3200-3400 cm^-1^ showing the presence of OH stretching and N-H stretching vibrations in the N-acetyl side chain. The amide I group of C=O carboxyl and C-O-C stretching are ascribed to the peaks at 1600-1660 cm^-1^ and 1035 cm^-1^, respectively 41-43. Similar peaks were detected in HA-GMA, suggesting that no significant chemical alterations occurred after the reaction. The spectra of gelatin reveal the presence of amide I (1600-1660 cm^-1^, associated with C=O stretching) and amide II (1470-1570 cm^-1^, involving C-N stretching vibrations in combination with N-H bending). Additionally, a broad peak at 3200-3400 cm^-1^ is observed, indicating OH stretching and N-H stretching in peptide bonds 44, 45. These peaks appeared in Gel-GMA, indicating no chemical changes in the amide bonds following the reaction. Specially, HA-GMA and Gel-GMA showed small peaks around 950 and 1490 cm^-1^ (C=C) indicating methacrylation reaction via the GMA 46, 47. GH-GMA exhibits similar peaks to Gel-GMA, likely due to a higher proportion of Gel-GMA in the GH-GMA composites. However, the C-O-C stretching peak (1035 cm^-1^) in GH-GMA increased compared to Gel-GMA. Weak peaks indicative of methacrylate carbon-to-carbon double bonds still appeared after mixing Gel-GMA and HA-GMA. This indicates that GH-GMA incorporates the characteristics of both HA and gelatin while introducing a photo-curable moiety. The amorphous nature and crystallinity of the materials were assessed through XRD analysis of gelatin, HA, Gel-GMA, HA-GMA, and GH-GMA, as shown in **Figure 1F** and **Figure S2**. Both gelatin and HA primarily exhibited amorphous characteristics. However, following the conjugation of GMA, the broad peak at 2θ = 20° for gelatin and the small, broad peak at 2θ = 10° for HA were reduced, suggesting that GMA conjugation enhances the amorphous nature of the macromolecules. The Gel-GMA and HA-GMA mixtures also predominantly displayed amorphous characteristics. These enhanced amorphous properties are expected to offer advantages in water absorbency, photo-reactivity, and processability due to improved flexibility, making them suitable materials for photo-crosslinkable spheroid fabrication in this study.

**Figure 2:**
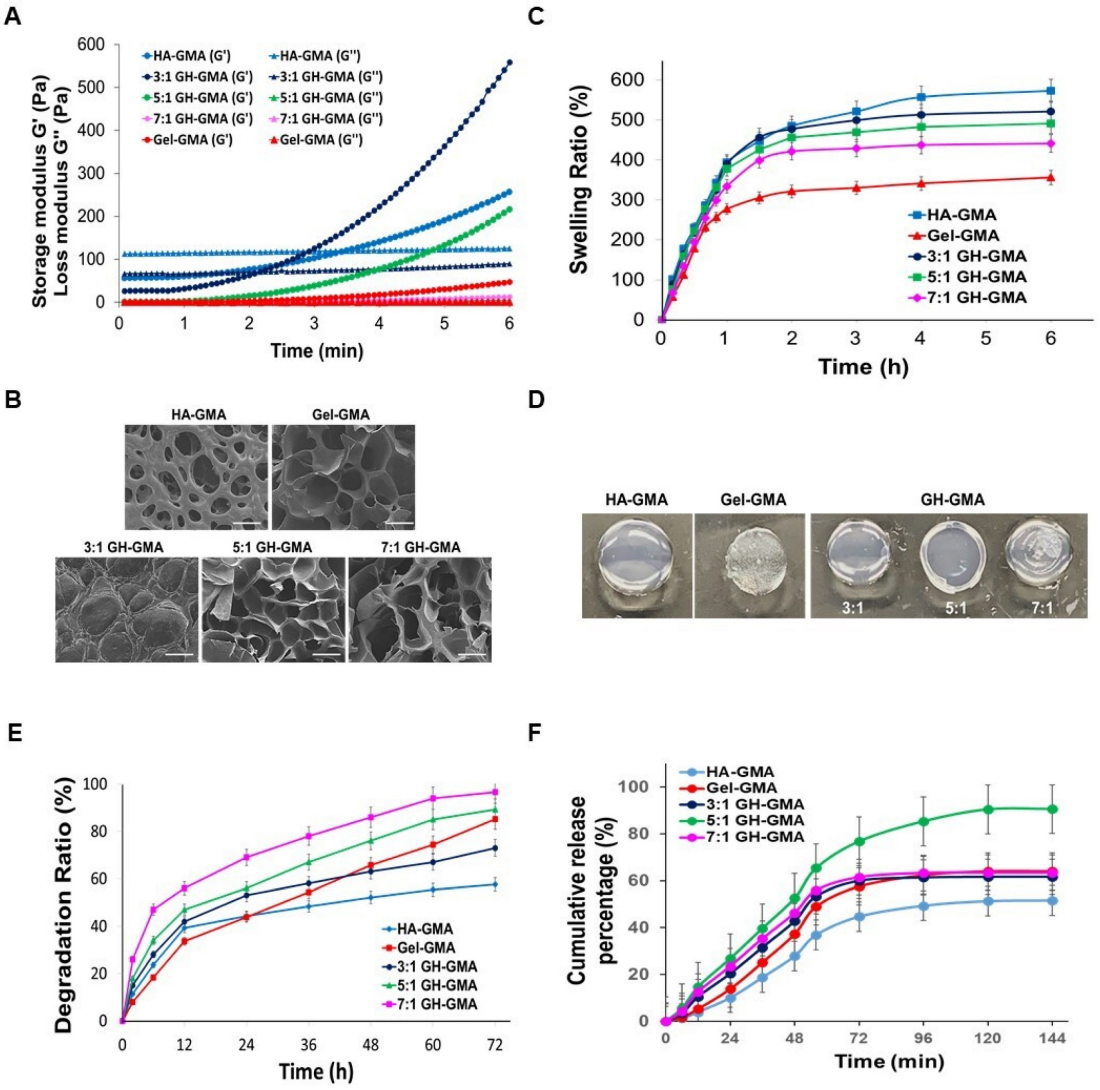
** Characterization of GH-GMA hydrogel. (A)** Rheological analysis: Comparison of storage (G') and loss (G'') modulus using a rheometer of pre-hydrogels **(B)** Scanning electron microscopic (SEM) findings (Scale bar showed 100 µm), **(C)** *in vitro* swelling test in PBS (pH7.0) for 6 h, **(D)** swollen status at 1.5 h after immersed in PBS, **(E)** *in vitro* degradation of hydrogels in PBS contained 40 mU/ml of hyaluronidase for 72 h, and **(F)** IL-2 cytokine release test by ELISA of hydrogels. 10% GH-GMA composites at different compositions (3:1, 5:1, and 7:1 of Gel-GMA and HA-GMA) were compared with 10% Gel-GMA and 5% HA-GMA) in all tests. Three independent experiments were performed in all analysis.

The rheological properties of the GH-GMA hydrogels were monitored during the UV-curing process to assess the kinetics of the photo-crosslinking reaction (**Table 2 and Figure 2A**). The storage modulus (G') and loss modulus (G'') represent the elastic part and viscous parts of hydrogels, respectively. G' was larger than G'' in all groups, regardless of components. Hydrogels tended to become stiffer with increasing curing times, indicating that longer exposure to UV light led to greater stabilization of the hydrogel. The time for the gel point which indicates a crossover for G' and G'' 48, showed a decreasing trend for G' in the order of 3:1, 5:1, and 7:1 GH-GMA. Complex viscosity is a measure of the resistance to deformation when shear stress is applied and is one of the factors used to determine needle injectability 49. The complex viscosity decreased with the addition of Gel-GMA. Specifically, the complex viscosity at the initial stage in the 3:1 GH-GMA group was 190 times higher than in the 7:1 GH-GHA and 127 times higher than in the Gel-GMA group itself, which is derived from the HA. J. Picard *et al*. observed in the liquid state, the polysaccharide dictates the rheological properties of the protein-polysaccharide mixture, while gelatin influences viscosity quantitatively. HA predominantly exists in the sol phase of the protein gel, inducing an entangled state that impacts the overall viscoelasticity of the system 50. The complex viscosity exhibited a significant difference that persisted even after full crosslinking because Gel-GMA provided entanglements to the HA-GMA, enhancing viscoelasticity. High viscosity in the mixing process of hydrogel precursor with cells can expose cells to damaging shear forces, leading to decreased cell viability. In contrast, low-viscosity solutions reduce shear forces, enhancing cell viability during mixing 51. A Eddhahak *et al.* reported that hydrogels with lower viscosity are more conducive to cell proliferation and preserving cell phenotype. The reported threshold value for complex viscosity to achieve 90% cellular viability was 91.5 Pa·s 52. All GH-GMA composites showed values below this threshold within 6 min since photo-crosslinking.

Open and interconnected macroporous structures in a hydrogel scaffold are vital for smooth cell penetration, unrestricted cell growth, ECM secretion, and effective transport of oxygen, waste products, and nutrients 53. The cross-sectional morphologies of hydrogels were observed using a scanning electron microscope (SEM) (**Figure 2B**). The photo-polymerized Gel-GMA and HA-GMA hydrogel exhibited entirely different microscopic morphologies. In the GH-GMA composites, as the concentration of Gel-GMA increased, the freeze-dried hydrogel revealed highly porous microstructures with an interconnected open pore morphology, resembling the structure of Gel-GMA. These results demonstrate that the ratio of Gel-GMA to HA-GMA significantly influences the formation of hydrogel microstructures and will affect to cell growth.

A swelling assay was conducted on all photo-polymerized hydrogels in PBS (pH 7.0) for 6 h (**Figure 2C**). In all groups, most of the water absorption occurred within the first 1.5 h, and reached a constant value after 5 h. The HA-GMA hydrogel exhibited the highest swelling ratio among the groups, during the test. The swelling characteristics of the hydrogel based on GH-GMA composites decreased in a manner similar to that of Gel-GMA hydrogel, depending on the increasing content of Gel-GMA in the composites. After reaching equilibrium (1.5 h) in PBS (**Figure 2D**), the shape and color of each hydrogel changed, the swollen size of hydrogel was decreased with an increase in the Gel-GMA components in the GH-GMA. The degradation studies mimic the potential breakdown of the hydrogels *in vivo*, affirming that the endorsed biodegradable scaffold is suitable as a carrier for cells. *In vitro* degradation rate of GH-GMA composites was compared with Gel-GMA and HA-GMA in PBS (pH 7.0) which contained 40 mU/ml of hyaluronidase up to 72 h (**Figure 2E**). The HA-GMA hydrogel underwent slow degradation compared to other groups. As the Gel-GMA ratio in the composites increased, the degradation ratio increased. This can be understood as an increase in the surface area available for the enzyme to access due to the decrease in HA content and increase in gelatin content within the hydrogel. As the amount of gelatin increases, the HA network loosens and porosity rises, facilitating easier enzyme penetration. The degradation of HA reduces the hydrogel's mechanical strength and stability, leading to a faster overall degradation.

IL-2 plays a pivotal role as an essential element in the proliferation and activation of CD8^+^ T cells. Its critical function in immune system activation within the TME presents a promising avenue for achieving comprehensive cancer eradication 54, 55. IL-2 cytokine release curves were generated to quantify the IL-2 released from hydrogels by ELISA (**Figure 2F**). The graph shows that IL-2 was released more rapidly and accumulated to a higher level in the GH-GMA composite-based hydrogels compared to the Gel-GMA and HA-GMA-based hydrogel in the initial stage. However, the figures were not significantly different. Among the GH-GMA composites, the hydrogel composed of Gel-GMA and HA-GMA in a 5:1 ratio exhibited a gradual and sustained release of IL-2, with the highest cumulative amount compared to the other GH-GMA hydrogels. On the other hand, the other hydrogels released IL-2 rapidly, reaching a maximum release at 48 h, and then the release rate dramatically decreased. We believe that the drug encapsulation and gelation properties of the hydrogel influence drug release, with the 5:1 combination providing a balanced outcome. Positively charged IL-2 interacts more with the carboxyl and hydroxyl groups of HA, so as the HA concentration decreases, it can release more quickly and in greater amounts. However, the high crosslinking density of Gel-GMA also impacts IL-2 release. In the 7:1 GH-GMA, the increased gelatin content results in higher crosslinking density, making the hydrogel firmer and reducing swelling, which in turn is expected to slow down the release of IL-2. Based on our findings, we confirmed the adjustability of properties based on the composition of gelatin and HA. The 5:1 GH-GMA group, which exhibited intermediate values across various physical properties, is considered favorable for CD8^+^ T cell cultivation due to its highest cumulative release of IL-2 cytokines. Consequently, 5:1 GH-GMA was chosen as the primary material for subsequent experiments.

### Characterization of human tonsil-derived mesenchymal stem cells (TMSCs)

**Figure 3:**
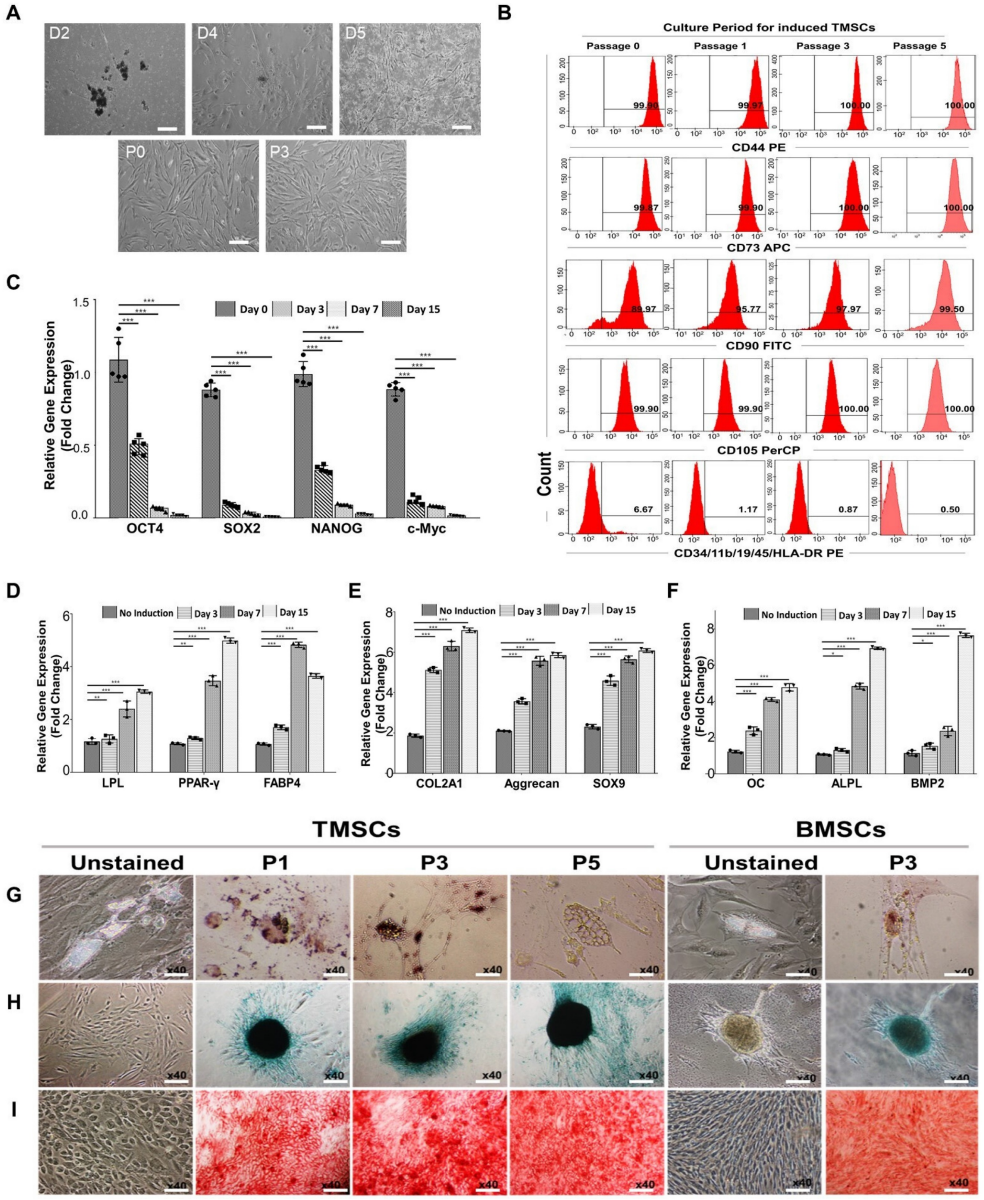
** Characterization of human tonsil-derived mesenchymal stem cells (TMSCs). (A)** Morphological changing of TMSCs were observed in a passage dependent manner using inverted microscope (x40), scale bar=500 μm (see **Figure S1** for the flow chart). **(B)** Immunophenotypic changes of TMSCs with passage dependent analysis. Specific antigenicity of TMSCs were characterized and analyzed with mesenchymal stem cell-specific surface markers (CD44, CD73, CD90, and CD105), and haematopoietic & immune cell markers (CD34, CD11b, CD19, CD45, and CD HLA-DR) using flow cytometer. **(C)** qRT-PCR. Stemness confirmation of induced TMSCs with specific gene expression (OCT4, SOX2, NANOG, and c-Myc) by qRT-PCR from passage dependent. Non-induced TMSCs were used as a control. (see **Figure S3A** for gel electrophoresis).** (D-F)** qRT-PCR. After trilineage differentiation of TMSCs, specific gene expression was compared at the day 3 (passage no 1), day 7 (passage no 2), and day 15 (passage no 4) compared to non-induced TMSCs as a control. (**D**) LPL, PPAR-γ, and FABP4 involved in adipogenesis, (**E**) COL2A1, aggrecan, and SOX9 are involved in chondrogenesis, and (**F**) OC, ALPL, and BMP2 involved in osteogenesis. (see **Figure S3B** for gel electrophoresis). Gene expression levels were normalized to that of GAPDH, and showed in fold changes as marker gene vs GAPDH ratio. **(G-I)** Immunostaining. On the 21st day of the induction culture, trilineage differentiation of TMSCs at passages no 1, 3, and 5 was compared with BMSCs (passage no 3). Evaluation of (**G**) adipogenic differentiation by Oil Red O staining to visualize lipid accumulation, (**H**) chondrogenic differentiation potential by Alcian blue staining to see glycosaminoglycan, and (**I**) osteogenic differentiation by Alizarin red staining to visualize calcium deposition. Magnification x40. Scale bar = 500 μm. Three independent experiments were performed in all analysis. Data are expressed as the means ± S.D. of triplicate samples. *p < 0.05, **p < 0.01, ***p < 0.001.

For the isolation of tonsil cells, enzymatic digestion and density gradient methods were used. After isolating and plating of tonsil cells obtained from the tonsil tissue, these cells successfully attached to the culture plate within 2 days. They formed colony-forming unit fibroblast (CFU-F) upon adhering to the culture plate at day 4. These colonies were subsequently expanded, and spindle-shaped cells grew, resulting in the formation of a homogenous monolayer of adherent fibroblastic cells (**Figure 3A**). On day 7 of cultivation from the initial plating, the tonsil-derived cells were sub-cultured when they reached 80% confluency. This sub-culturing process was continued until passage 5 to obtain tonsil mesenchymal stem cells (TMSC), and an immunophenotypic analysis of TMSCs was performed in a passage-dependent manner. TMSCs at passage 0, 1, 3, and 5 were evaluated using a flow cytometer to assess the expression of mesenchymal specific markers (CD44, CD73, CD90, and CD105), as well as haematopoietic and immune cell markers (CD34, CD11b, CD19, CD45, and HLA-DR) for negative expression (**Figure 3B**). The results revealed that the induced TMSCs were strongly positive for all MSC-specific markers (99.9% for CD44, 99.87% for CD73, 89.97% for CD90, and 99.9% for CD105), starting from passage 0. The expression rate of each MSC-specific marker reached almost 100% at passage 5 (100% for CD44, CD73, and CD105, and 99.50% for CD90). However, the antigenicity for CD34, CD11b, CD19, CD45, and HLA-DR gradually decreased depending on the passage periods, with the expressed population being 6.67% at passage 0, 1.17% at passage 1, 0.87% at passage 3, and 0.50% at passage 5. This pattern of MSC-specific immunophenotype expression was similar to characterized bone marrow-mesenchymal stem cells (BMSCs) 56, 57.

The expression of stemness markers in the induced TMSCs was examined including octamer-binding transcription factor-4 (OCT-4), sex determining region Y-box 2 (SOX-2), NANOG, and c-Myc gene (**Figure S3A and Figure 3C**), which function in stem cell self-renewal or pluripotency maintenance in stem cells 58. The gel electrophoresis results indicate the detection of all genes in TMSCs across all passages. Simply, the expressed bands were strongest at passage 0, gradually diminishing over the culture periods. qRT-PCR results, expressed as fold changes for each culture period, demonstrated a significant decrease in all genes starting from culture day 3 after TMSCs induction.

For the investigation the differentiation potential of TMSCs depending on induction periods, TMSCs at induction day 0, 3, 7, and 15 were differentiated toward adipogenesis, chondrogenesis, and osteogenesis. This tri-lineage differentiation capacity was confirmed by qRT-PCR and gel electrophoresis. For adipogenesis, the genes lipoprotein lipase (LPL), peroxisome proliferator-activated receptor gamma (PPAR-γ), and fatty acid binding protein 4 (FABP4) were analyzed. For chondrogenesis, the genes collagen type II alpha 1 chain (COL2A1), SRY-box transcription factor 9 (SOX-9), and aggrecan were examined. Lastly, for osteogenesis, the genes osteocalcin (OC), alkaline phosphatase (ALP), and bone morphogenetic protein 2 (BMP2) were studied (**Figure S3B and Figure 3D-F**). Non-induced TMSCs (Day 0) were used as a control. Interestingly, in contrast to the expression pattern of stemness markers, tri-lineage-specific genes were gradually and strongly expressed as the induction period increased. To further investigate the differentiation potential of TMSCs depending on passage periods, TMSCs at passage 1, 3, and 5 underwent adipogenesis, chondrogenesis, and osteogenesis for 21 days (**Figure 3G-I**). The cells were stained using special staining and compared to BMSCs at passage 3. Adipogenic differentiation was assessed by detecting the accumulation of cytoplasmic lipid vacuoles using the Oil Red O staining method. In both groups, many cells were observed with large and round lipid bodies in their cytoplasm, and stained lipid vacuoles were detected at all passage periods. Chondrogenesis was conducted using the hanging-drop culture method and verified by Safranin O staining. After differentiation, chondrogenic colonies were detected in both groups. Osteogenic differentiation was confirmed by Alizarin Red staining. In both TMSCs and BMSCs, intense and widespread staining revealed deposition of calcium-rich extracellular matrix. Mesenchymal stem cells (MSCs) are multipotent stromal cells and are the primary material used to induce cells to undergo lineage-specific differentiation. They can differentiate into chondrogenic, osteogenic, and adipogenic lineages 59. The results above demonstrate the differentiation potential of TMSCs and indicate that their ability to differentiate into osteoblasts and adipocytes is comparable to that of BMSCs across these three differentiation pathways.

### Tonsil derived lymphatic endothelial cells (T-LEC) and spheroids formation

TMSCs were induced to differentiate into lymphatic endothelial cells (T-LECs), and their properties were characterized. For the differentiation towards LECs, TMSCs at passage 3 were cultured in EGM-2MV medium supplemented with 100 ng/ml VEGF-C for 20 days. VEGF-C, a key factor in lymphatic vessel growth, has been found to improve edema resolution by fostering the development of functional capillaries 60. Moreover, it facilitates the proliferation of CD8^+^ T cells activated by anti-CD3 and anti-CD28 stimuli 61.

**Figure 4:**
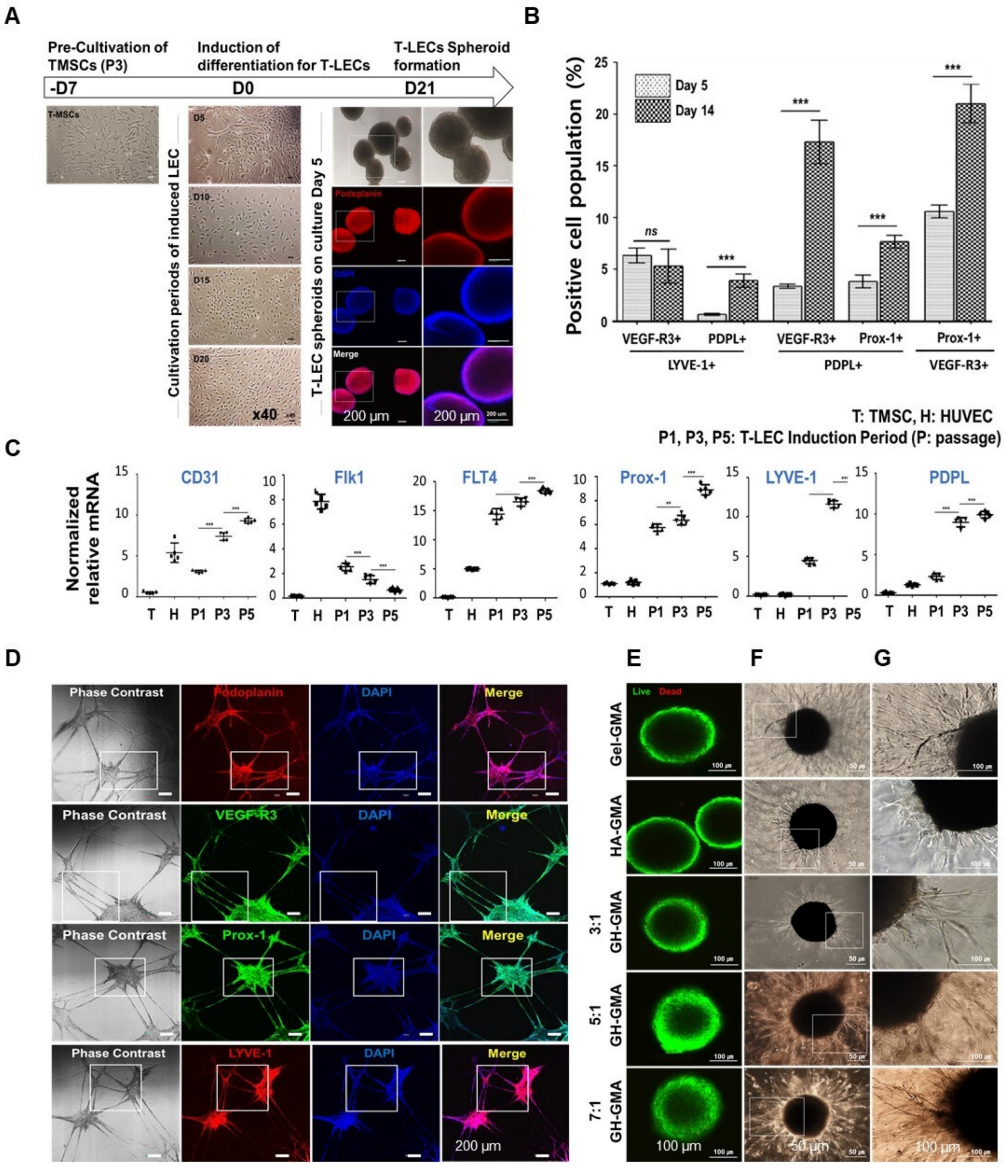
** Evaluations of tonsil derived lymphatic endothelial cells (T-LEC) and T-LEC based spheroids. (A)** Development of T-LECs and formation of T-LEC spheroids. (*l*) Inverted microscopic observation was performed cultivation day at 5, 10, 15, and 20 compared to non-induced TMSCs. (*r*) Generated T-LEC spheroids were stained with lymphatic specific marker, podoplanin and DAPI for nuclear at induction day 5. Scale bar= 200 µm. **(B)** Flow cytometry quantification. Generated T-LEC at day 5 and day 14 were analyzed using flow cytometer stained with LEC specific markers, VEGF-R3, Prox-1, LYVE-1, and PDPL. (See **Figure S4A** for their plots). **(C)** qRT-PCR. Lymphatic specific gene expression in induced T-LECs were analyzed using VEGF-R3, Prox-1, LYVE-1, and PDPL compared with CD31 and Flk1 genes. HUVEC and non-induced TMSCs were used as controls. (See **Figure S4B** for the result of gel electrophoresis)** (D)** Lymphatic network formation using Matrigel^TM^ of induced T-LECs and confirmation of their specific phenotypes with podoplanin (PDPL), VEGF-R3, LYVE-1, and Prox-1 by immunofluorescent staining. DAPI (blue) used for nuclear staining. Scale bar = 200 µm. (See **Figure S4C** for enlarged images of small boxes highlighted in Figure 4D) **(E-G)** Characteristics of T-LEC spheroids encapsulated in the hydrogels. T-LEC spheroids were encapsulated in 10% Gel-GMA, 5% HA-GMA, three 10% GH-GMA composite-hydrogels supplemented with VEGF-C for 3 days. (**E**) Cell viability test using Live & Dead assay with Calcein AM (live cells, green) and ethidium homodimer-1 (dead cells, red) in the hydrogel. Scale bar = 100 µm. (**F**) Lymphatic sprouting activity of induced T-LEC spheroids. (**G**) Enlarged area of the T-LEC spheroid that is highlighted in (F). Scale bar = 50 µm (**F**) and 100 µm (E and G), respectively. Experiments were performed three times replicate. Data are expressed as the mean ± S.D. *p < 0.05, **p < 0.01, ***p < 0.001. ns = no significant for Figure 4B-C.

Morphological changes were observed using an inverted microscope (**Figure 4A(*l*)**). The morphologies of TMSCs gradually changed from a fibroblastic spindle-shaped to an oval shape up to induction day 20. Moreover, a cobblestone area appeared at induction day 15, similar to that of endothelial cells 62. T-LEC spheroid was generated with induced T-LECs at day 21 using an ultra-low attached 96-well microplate to achieve uniform size formation. The harvested T-LEC cells at 5 x 10^3^ cells/200 μl in EGM-2MV with 500 ng/ml VEGF-C were cultured for 5-7 days. Immunofluorescent detection with the lymphatic endothelial-specific marker, podoplanin, was performed (**Figure 4A(*r*)**). T-LEC spheroids on day 5 were strongly stained with podoplanin.

For the confirmation of lymphatic specific antigenicity, induced T-LEC single cells were double stained with lymphatic specific antibodies: Prospero homeobox 1 (Prox1), lymphatic vessel endothelial hyaluronan receptor 1 (LYVE‑1), vascular endothelial growth factor receptor‑3 (VEGFR‑3) and podoplanin (PDPL), and then analyzed using flow cytometric analysis. Unstained TMSCs were used as a negative control. The double positive population was compared at induction day 5 and day 14 (**Figure S4A and Figure 4B**). Double positive population were 10.60 ± 0.6%, 21.03 ± 1.8% for VEGF-R3^+^ Prox-1^+^, 6.38 ± 0.7%, 5.38 ± 1.6% for VEGF-R3^+^ LYVE-1^+^, 3.825 ± 0.6%, 7.675 ± 0.5% for PDPL^+^ Prox-1^+^, 0.65 ± 0.1%, 3.925 ± 0.6% for PDPL^+^ LYVE-1^+^, 3.375 ± 0.2%, 17.33 ± 2.0% for PDPL^+^ VEGF-R3^+^ at induction day 5 and day 14, respectively. PDPL^+^ LYVE-1^+^, Prox-1^+^PDPL^+^, and Prox-1^+^VEGF-R3^+^ populations were gradually increased depending on induction periods, whereas VEGF-R3^+^ LYVE-1^+^ population showed a slight decrease.

Additionally, qRT-PCR was conducted to confirm the specific gene expression of induced T-LECs (**Figure S4B and Figure 4C**). Non-induced TMSCs and HUVEC cells were used as controls for comparison. The results showed that CD31 (Cluster of Differentiation 31), general endothelial cell marker, was gradually expressed on T-LECs, similar to HUVEC, and its expression increased with induction periods. On the other hand, Flk1, vascular endothelial growth factor (VEGF) receptor 2, was strongly expressed in HUVEC, but its expression gradually decreased in T-LECs during the induction period. Regarding lymphatic specific genes, FLT 4 (VEGF-R3), Prox-1, LYVE-1, and PDPL were remarkably increased in induced T-LECs depending on induction periods. When compared to TMSCs or HUVEC, the expression levels of these lymphatic-specific genes were significantly higher in T-LECs. These findings confirm that the induced T-LECs exhibited specific gene expression profiles characteristic of lymphatic endothelial cells, distinguishing them from non-induced TMSCs and HUVEC cells.

The tube formation assay was conducted to assess the functional capability of induced T-LECs. The cells were then identified using immunofluorescent staining with specific antibodies: podoplanin, VEGFR-3, Prox1, and LYVE-1 (**Figure 4D and Figure S4C**). Following the seeding of induced T-LECs at passage 3 on Matrigel, the neovessel formation was observed. Furthermore, the interconnection of these vessel branches was detected. Immunofluorescence staining results revealed strong staining of LEC-specific antibodies on the neovessels. These findings suggest that multipotent TMSCs can be successfully differentiated into lymphatic endothelial cells. Notably, their functional abilities and specific gene expressions increased in correlation with the duration of the induction period.

To determine the cell proliferation and viability, 5 days-induced T-LEC spheroids were encapsulated with 10% Gel-GMA-, 5%HA-GMA-, 10% 3:1, 5:1, and 7:1 GH-GMA composites-based hydrogels. The encapsulated T-LEC spheroids were cultured for 72 h. The proliferation activity of each hydrogel encapsulated T-LEC spheroids showed no significant differences among various hydrogels (**Figure S4D**). Confocal images after Live/Dead assay revealed cell viability of T-LEC spheroids encapsulated in each hydrogel (**Figure 4E**). In the result, first of all, confocal images showed that round shape of T-LEC spheroid is maintained for 72 h. Dead cells rarely found in all of hydrogel combination. Also, live cells in green fluorescence and dead cells in red fluorescence showed no differences among groups. These results indicate that various concentration of mechanically mixed GH-GMA proposed good cell proliferation ability.

The sprouting ability of T-LEC spheroids encapsulated in various hydrogels was assessed using single T-LEC spheroids in EGM-2MV media supplemented with 100 ng/ml VEGF-C for 72 h (**Figure 4F-G**). In the overall observation, it was evident that 10% Gel-GMA exhibited the most extensive sprouting formation. T-LEC spheroids in 10% 5:1 and 7:1 GH-GMA composites-based hydrogels exhibited a considerable increase in size, accompanied by the development of longer and denser vessel branches compared to those within the 5% HA-GMA and 10% 3:1 GH-GMA hydrogels. These results suggest that the presence of gelatin in the GH-GMA composites significantly influences the sprouting ability of T-LEC spheroid within a 3D hydrogel environment.

### Stimulation of T-CD8^+^ T cells (STCs)

While CD4^+^ T cells aid in coordinating immune responses, the immunological functions of CD8^+^ T cells are primarily executed through their capacity to selectively release cytolytic molecules, perforin, and granzymes, leading to the destruction of infected or aberrant cells. Additionally, they can secrete cytokines like IFN-γ and IL-2, further enhancing their immune response capabilities 63, 64. IL-2 is a pleiotropic cytokine crucial for regulating the differentiation and homeostasis of pro- and anti-inflammatory T cells. It also plays a significant role in the development of CD8^+^ effector cells. IL-2 secretion and subsequent signaling through the IL-2 receptor are stimulated by CD28 co-stimulation, which enhances T cell expansion, making IL-2 the preferred cytokine for T cell culture 65-67.

**Figure 5:**
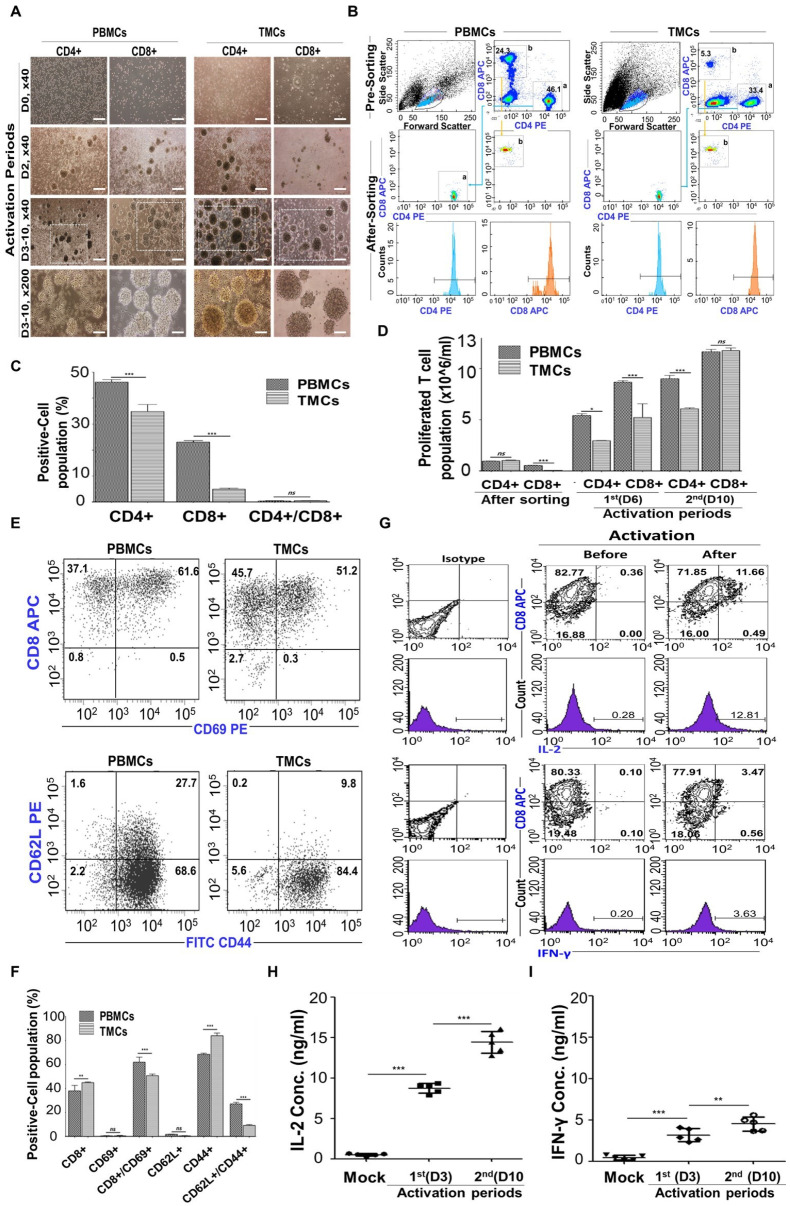
** Preparation and Characterization of stimulated CD8^+^ T cells (STCs). (A)** Morphologies during activation and expansion of sorted tonsil derived CD4^+^ and CD8^+^ T cells by anti-CD3 and anti-CD28 antibodies supplemented with IL-2 up to 10 days cultivation. Scale bars= 500 μm (x40) and 200 μm (x100) (See **Figure S5-6** for more details)** (B-D)** Flow cytometry. **(B)** CD4^+^ or CD8^+^ T cell populations before sorting by flow cytometer. **(C)** Quantification of cell populations. (**D**) Proliferated T cell population after both sorting and 2 steps activation. **(E-F)** Double color analysis CD8^+^ T cells from PBMC and tonsils after 3 days activation. Each cell was stained with anti-CD8 APC/anti-CD69 PE and anti-CD62L PE/anti-CD44 FITC monoclonal antibodies. (**E**) Plots and **(F)** quantification of flow cytometer. (**G-I**) The secretion of intracellular cytokines, IL-2 and IFN-γ, by STCs **(G)** Plots after double staining using flow cytometry. **(H-I)** ELISA. **(H)** IL-2 and **(I)** IFN-γ. Mock samples were utilized as controls, representing the unstimulated CD8^+^ T cells. Experiments were performed three times replicate. Data are expressed as the mean ± S.D. *p < 0.05, **p < 0.01, ***p < 0.001.

To make activated CD8^+^ T cells, we subsequently collected non-adherent cells (TMCs) after plating tonsil-derived cells to purify the CD8^+^ T cell population. **(Figure S5)**. Peripheral blood mononuclear cells (PBMCs) were used as a control. The non-adherent cell population were cultured for 24 h before being sorted using a flow cytometer. Morphological analysis of each T cell population revealed that CD4^+^ and CD8^+^ T cells from tonsils exhibited very similar morphology and colony formation compared to PBMCs (**Figure S6 and Figure 5A**). The sorted CD4^+^ and CD8^+^ populations from PBMCs and TMCs exhibited proportions of 46.1 ± 1.34 % and 33.4 ± 0.67% for CD4^+^, 24.3 ± 0.77% and 5.3 ± 0.26% for CD8^+^, respectively (**Figure 5B-C**). Following sorting, the purity of each population was 99.3 ± 0.15%. Subsequently, the sorted CD4^+^ and CD8^+^ T cells were cultured for up to 10 days with anti-CD3 (as a TCR stimulus) and anti-CD28 (as a costimulatory cue) antibodies to facilitate proliferation and expansion, while exogenous IL-2 supplementation was used to promote T cell activation (**Figure 5D**). After 6 days of stimulation, the numbers of CD4^+^ and CD8^+^ T cells from both PBMCs and TMCs increased compared to their initial activation state. Moreover, after 10 days of stimulation, each T cell population from the tonsils exhibited a remarkable increase compared to those from PBMCs.

CD69 plays various roles in T cell activation, migration, and immune responses, making it a promising target for immunotherapy and prognostic prediction due to its dynamic expression and interactions 68. CD44 acts as a prominent activation marker, distinguishing effector and memory T cells from naïve ones. It plays critical roles in cell adhesion, migration, and tissue homing by binding to hyaluronic acid. Additionally, CD44 is upregulated during T cell activation and differentiation 69. CD62L, initially expressed on naïve T cells, undergoes downregulation upon activation, indicating differentiation into effector memory T cells 69. After 3 days of stimulation, expression of CD8/CD69 and CD62L/CD44 were detected in CD8^+^ T cells to confirm T cell activation and differentiation (**Figure 5E-F**). A similar expression patterns of CD8/CD69 and CD62L/CD44 were observed in both groups. TMCs and PBMCs showed highest levels of CD8^hi^CD69^hi^ that were expressed by 51.2% of TMCs derived CD8^+^ T cells *vs.* 61.6% of PBMCs derived CD8^+^ T cells in the panel of CD8/CD69. Both groups also showed highest levels of CD62L^lo^CD44^hi^ phenotype (84.4% of TMCs derived CD8^+^ T cells and 68.6% of PBMCs derived CD8^+^ T cells) in the panel of CD62L/CD44. These findings suggest that STCs represent characteristics of activated effector memory CD8^+^ T cells, which resulted from activation process including IL-2 70. Hence, the CD62L^lo^CD44^hi^ phenotype denotes activated effector memory CD8^+^ T cells isolated from tonsils, proficient in prompt immune responses and adept at eliminating infected or malignant cells 71.

Upon infection or by APCs, naive antigen-specific CD8^+^ T cells differentiate into cytotoxic T lymphocytes (CTLs) that produce proinflammatory cytokines like IL-2 and IFN-γ, which are vital for controlling CD8^+^ T cell expansion and contraction during immune responses to intracellular pathogens, and they gain the ability to eliminate infected cells 54, 72, 73. To see that our CD8^+^ T cells were able to produce cytokines IL-2 and IFN-r after activation by anti-CD3 and anti-CD28 along with exogenous IL-2, intracellular cytokines IFN-γ and IL-2 secreted by activated STCs were detected depending on the duration of activation using flow cytometer with double staining (**Figure 5G**) and ELISA (**Figure 5H-I**). After 3 days of activation, the concentrations of IL-2 and IFN-γ increased, particularly with IL-2 exhibiting higher levels of secretion across a wider range compared to mock condition (unactivated T-CD8^+^ T cells).

### Co-encapsulation of induced T-LEC spheroid and STCs

**Figure 6:**
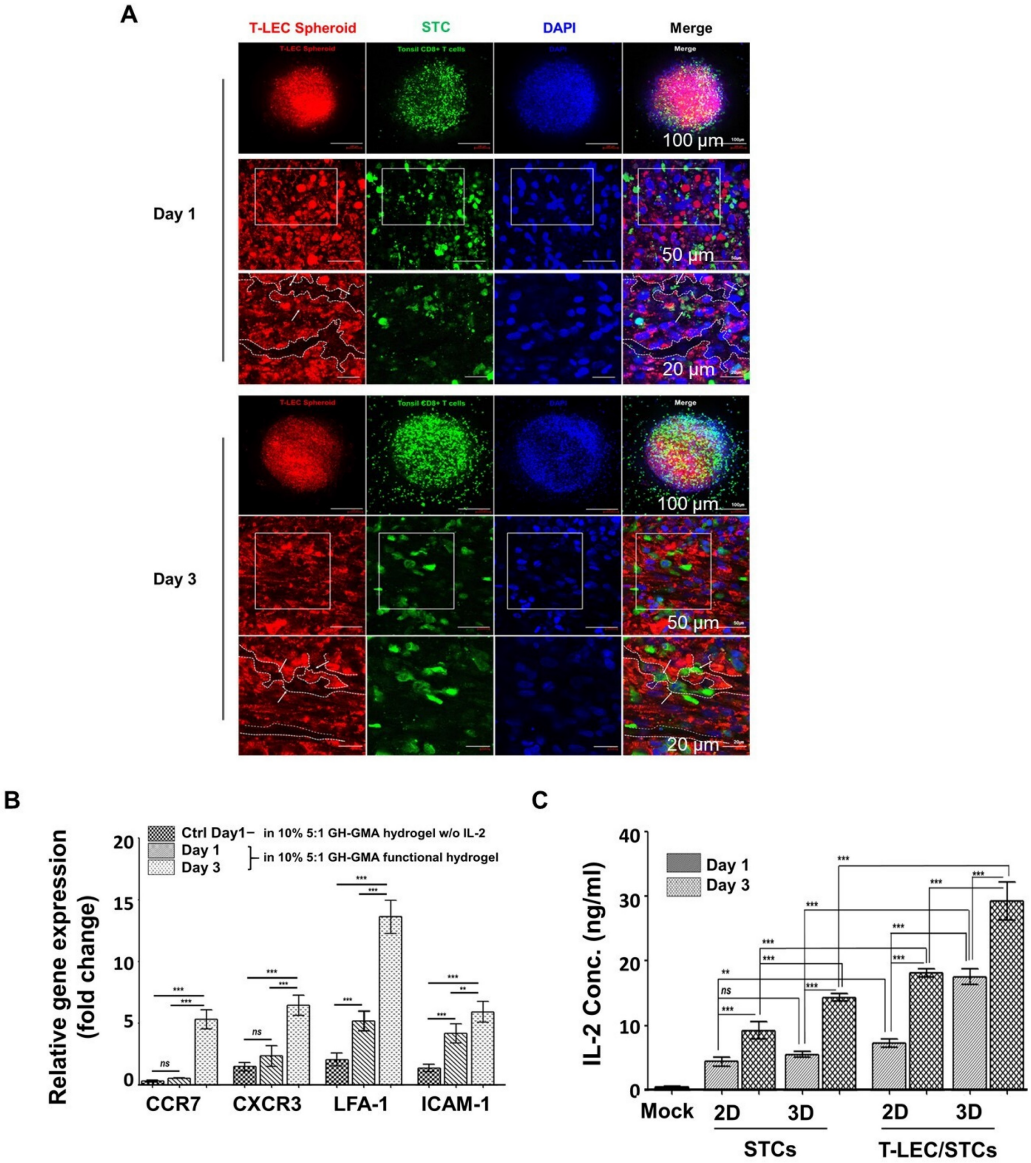
** Co-encapsulation of induced T-LEC spheroid and STCs in the functionalized hydrogel. (A)** Co-encapsulation of induced T-LEC spheroid and STCs for 1 day and 3 days cultivation in 10% 5:1 GH-GMA hydrogel supplemented with IL-2 and VEGF-C. T-LEC spheroid was stained with podoplanin (red), CD8^+^ T cell with CD8 (green) and nucleus with DAPI (blue). Scale bars = 100 µm, 50 µm and 20 µm, from top to bottom. **(B)** T cell migration-related genes using quantitative RT-PCR. Encapsulated T-LEC spheroid and STCs in 10% 5:1 GH-GMA hydrogel supplemented IL-2 and VEGF-C was analyzed with CCR7, CXCR3, LFA-1, and ICAM-1 for T cell homing abilities. TMSCs in 10% 5:1 GH-GMA hydrogel without IL-2 used as a control. Data were quantified and normalized to the expression levels of GAPDH. **(C)** Quantitative analysis of IL-2 secretion from STCs co-culture with or without T-LEC spheroids. 2D liquid culture and 3D culture in 10% 5:1 GH-GMA hydrogel were compared. The mock group consisted of culturing only the functional hydrogel without cells. Each supernatant from culture condition were collected from the indicated cell cultures at day 1 and day 3. Experiments were performed three times replicate. Data are expressed as the mean ± S.D. (n=3, *p < 0.05, **p < 0.01, ***p < 0.001, and ns = no-significant).

The lymphatic endothelium plays an active role in modulating T cell responses, both through direct and indirect mechanisms 74. In this study, we hypothesize that LECs help maintain CD8^+^ T cell activation with the addition of exogenous antigens or stimulating cytokines using a biomimetic scaffold. To explore the interaction between T-LEC spheroids and STCs, T-LEC spheroids were labeled with red fluorescence and encapsulated together with green fluorescence-labeled STCs. This encapsulation was achieved using the functional hydrogel (10% 5:1 GH-GMA hydrogel enriched with anti-CD28, IL-2 and VEGF-C) to booster T cell activation in this study. The co-cultures were maintained for durations of 1 day and 3 days. The samples were subjected to analysis using confocal microscopy, with DAPI counterstaining for visualization (**Figure 6A**). After 1 day of co-cultivation, it became apparent that the structure of T-LEC spheroids was disrupted. Green fluorescence-labeled STCs began to infiltrate the areas of disruption within the red fluorescence-labeled T-LEC spheroid, as observed through Z-stack microscopy. By the third day of co-cultivation, a significant increase in disruption sites of the T-LEC spheroid was observed, accompanied by a higher concentration of STCs. These results illustrate that the co-culture model of T-LEC spheroids and STCs within the functionalized hydrogel facilitates the migration and invasion of CD8^+^ T cells into the induced lymphatic endothelial structure.

To confirm the migration of STCs, the expression of CD8^+^ T cell migration-related genes was assessed using qRT-PCR, targeting CCR7 (CC-Chemokine receptor 7), CXCR3 (C-X-C Motif Chemokines Receptor 3), LFA-1 (Lymphocyte Function-associated Antigen-1), and ICAM-1 (Intracellular Adhesion Molecules-1), within encapsulated T-LEC spheroids and CD8^+^ T cells cultured in the 10% 5:1 GH-GMA hydrogel with IL-2 (**Figure 6B**). Notably, the expression of CCR7, CXCR3, LFA-1 and ICAM-1 was found to be elevated after 3 days of encapsulation within the functional hydrogel, in comparison to the other culture duration and control (TMSCs encapsulated in 10% 5:1 GH-GMA hydrogel without IL-2). These results indicate that IL-2 loaded in GH-GMA hydrogel contributes to the sustained activation of CD8^+^ T cells and an enhanced acceleration of migration activity.

When T cells activated by LECs become apoptotic, exhaustion-associated factors like PD-1 and CD8 are upregulated, and the secretion of IFN-γ and IL-2 is reduced 75. To ascertain the impact of T-LEC spheroids when co-encapsulated with STCs within the functional hydrogel, the production of IL-2 was detected using ELISA (**Figure 6C**). This detection was conducted comparing the 2D/3D culture of STCs to the co-culture of STCs with T-LEC spheroids in both 2D and 3D culture groups. The mock group consisted of culturing only the functional hydrogel without cells. Culture supernatants were collected from both the 2D and 3D culture conditions after 1 day and 3 days. Upon comparing the 2D liquid culture with the 3D encapsulation involving STCs alone or in co-culture with T-LEC spheroids, it was evident that the IL-2 concentration increased in both 2D and 3D culture condition. Especially, a remarkable increase in IL-2 concentration was observed in the co-encapsulation of T-LEC spheroids with STCs within the GH-GMA hydrogel, surpassing the levels seen in any other culture condition. This is predicted to be the result of our functional hydrogel enhancing the interaction between STCs and T-LEC. Also, T-LECs affected IL-2 secretion from STCs.

The general paradigm for T cell activation necessitates three main signals: T cell receptor stimulation (signal 1), co-stimulation (signal 2), and pro-survival cytokines (signal 3) 31, 32, 76. These signals are typically provided by antigen-presenting cells (APCs) in the body. However, isolating natural APCs poses significant challenges, and maintaining stringent quality control is difficult. Moreover, replicating precise signaling pathways using artificial APCs remains complex, which ultimately restricts the expansion and functionality of T cells. These findings contribute to the understanding of LECs as APCs capable of cross-presenting exogenous antigens in conjunction with biomimetic hydrogels. It is known that LEC facilitates the proliferation of CD8^+^ T cells by collecting and presenting external antigens. Factors such as LFA-3 from lymph node-LEC can provide costimulation to enhance IL-2 secretion from activated T cells *in vitro* 77. On the other hand, there are also reports suggesting that LECs promote tolerance and prevent autoimmunity instead of activating immune responses 78, 79. In addition, it has been reported that LEC-induced activation of T cells may lead to abnormal changes and premature cell death 74. While our study highlights LEC's supportive role in T cell activation, further analysis of long-term T cell behavior is necessary. Nevertheless, the functional hydrogel containing LECs evidently promotes interactions with T cells under the experimental conditions.

### *Ex vivo* reconstituted 3D organotypic culture with cancer spheroids

**Figure 7:**
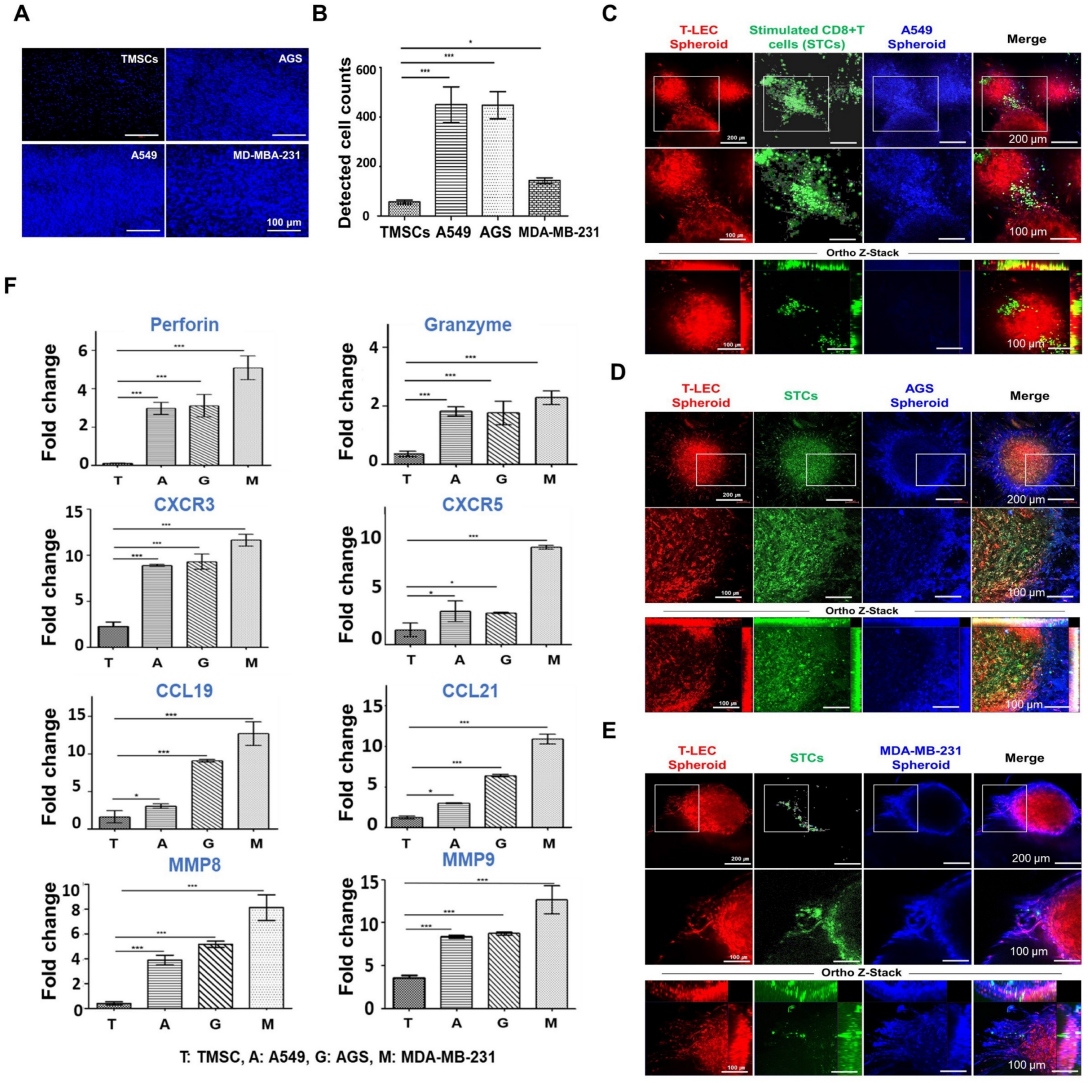
***Ex vivo* reconstituted 3D organotypic culture with cancer spheroids** (see **Figure S7** for experimental setting) **(A-B)** STCs migration assay (**A**) DAPI staining of STCs migrated from upper chamber, Scale bar = 100 µm. (**B**) Quantification of DAPI staining. Graph shows a comparison of migratory activity represented in the average number of transmigrated STCs per microscopic field of different cancer cell lines. **(C-E)** *Ex vivo* organotypic culture of (**C**) A549, (**D**) AGS, and (**E**) MDA-MB-231 cancer cell lines with encapsulated T-LEC and STCs in 10% 5:1 GH-GMA hydrogel for 3 days. Each cancer cell line was stained with podoplanin for T-LEC (red), CD8 for CD8^+^ T cell (green), and cell tracker violet (blue) for each cancer cell lines, and detected confocal microscope. Scale bar = 200 µm, 100 µm, and 100 µm from top to bottom. **(F)** Quantitative RT-PCR for *ex vivo* organotypic culture with cancer spheroids for 3 days. Perforin, Granzyme, CXCR3, CXCR5, CCL19, CCL21, MMP8, and MMP9 were quantified and normalized to the expression levels of GAPDH. Experiments were performed three times replicate. Data are expressed as the mean ± S.D. n=3, *p < 0.05, **p < 0.01, ***p < 0.001.

To create consistent cancer spheroids, ultra-low attachment 96-well plates were employed. Spheroids were cultivated for 7 days with A549 cells for lung cancer, AGS cells for gastric cancer, and MDA-MB-231 cells for triple-negative breast cancer, each cultured in the appropriate culture media for their respective cell lines. The resulting tumor spheroids had a diameter of 400-600 μm, with a cell dose of approximately 3 x 10^4^ per well following the 7-day cultivation period (**Figure S7A**). STCs migration was assessed against three cancer cell lines using a transwell assay (**Figure S7B**). The lower chamber of the transwell was seeded with each tumor spheroid, and TMSCs as a control, respectively. Transmigrated STCs were subsequently stained with DAPI (**Figure 7A-B**). A notable observation was that a higher number of migrated STCs were observed in co-culture with A549 and AGS cells in comparison to MDA-MB-231 and TMSCs. These findings highlight that the migration of STCs is influenced by the specific tumor cell types present, leads to act as a tumor infiltrating T cell population.

An *ex vivo* organotypic culture system with tumor cells was employed to replicate the TME consisted of cancer spheroids using 10% Gel-GMA and T-LEC spheroid and STCs encapsulated in 10% 5:1 GH-GMA loaded anti-CD28, IL-2 and VEGF-C. T-LEC spheroid and STCs encapsulated in 10% 5:1 GH-GMA were overlayed on cancer spheroids encapsulated in 10% Gel-GMA, which were cultured for 3 days (**Figure S7C**). Also, the approximate size of the 3D organotypic culture system was a thickness of 1 mm and a diameter of 5 mm. Each cell type was labeled with podoplanin for T-LEC (red), CD8 for CD8^+^ T cell (green), and three cancer cell lines (blue) (**Figure 7C-E**). The result revealed that the T-LEC spheroid and STCs complex exhibited a specific localization pattern, aligning themselves alongside each respective cancer spheroid. Notably, a subset of STCs was observed to migrate into the interior of the cancer spheroid. Furthermore, the cancer spheroids exhibited a remarkable stretching phenomenon, extending and branching into T-LEC formations, as evidenced by the z-stack imaging analysis. These results imply that STCs from the functional hydrogel encapsulated T-LEC could be migrated toward TME. To find deeper on the *ex vivo* organotypic culture with cancer spheroids, the gene expressions of Perforin and Granzyme (for cytotoxic CD8^+^ T cell activation), CXCR5 (for migration), C-C motif chemokine ligand (CCL)19 and CCL21 (for T cell proliferation and homing), and Matrix metalloproteinase (MMP)8 and MMP9 (for tumor infiltration) was assessed through qRT-PCR analysis (**Figure 7F**). Most of the gene expression in each *ex vivo* organotypic culture system with each cancer spheroid was increased. This indicates that STCs migrated from the functional hydrogels encapsulated LECs into the TME. Notably, the group composed of the malignant breast tumor, MDA-MB-231, demonstrated a markedly higher gene expression compared to both A549 and AGS across all the examined gene primers. These findings strongly indicate that our functional hydrogel encapsulated T-LEC and STCs were effectively activated within various malignancy-driven TMEs.

### Immune checkpoint PD-L1

Programmed death-ligand 1 (PD-L1) is expressed on tumor cells and induces immunosuppression to evade anticancer immune responses. It serves as a biomarker in various cancers, with its expression predicting disease outcomes 80. The programmed death 1 (PD-1)/ PD-L1 pathway plays a critical role as an immune modulator, particularly in its interaction with T-cells. PD-1 on T cells, acting as the receptor for PD-L1, negatively regulates cancer biology and promotes cancer progression and metastasis 81. Tumor cells with elevated PD-L1 expression levels demonstrate suppression of pathways associated with T-cell activation compared to cells with lower expression levels 82. The key molecules involved in T-cell regulation are strongly associated with the development of targeted therapies aimed at inhibiting PD-1 and PD-L1, thereby enhancing cancer immunotherapy.

**Figure 8:**
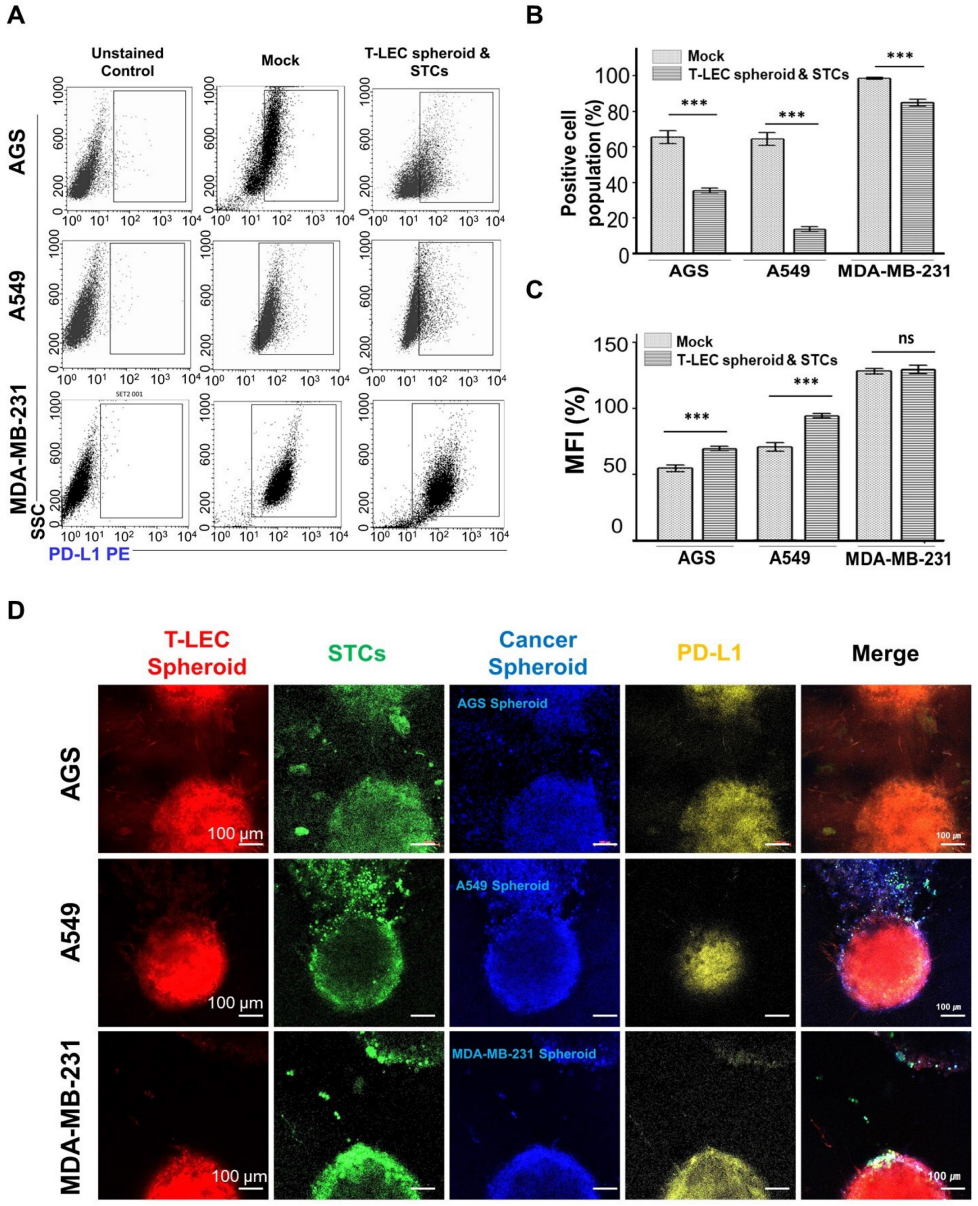
** PD-L1 expression from cancer cell lines under *ex vivo* organotypic culture.** Three cancer cell lines were encapsulated in a functional hydrogel (10% 5:1 GH-GMA hydrogel including IL-2 and VEGF-C) containing T-LEC and STCs.** (A-C)** Flow cytometry. (**A**) PD-L1 expression plots. (**B**) Quantification of PD-L1 positive cell population and (**C**) Mean Fluorescent Intensity (MFI) of PD-L1 positive cell population. Mock condition was cancer cell lines only. **(D)** Immunofluorescent staining of PD-L1 expressed on three cancer cell lines under *ex vivo* organotypic culture with functional hydrogel containing T-LEC and STCs. Scale bar = 100 μm (See **Figure S8** for enlarged images of small boxes highlighted in Figure 8D). Experiments were performed three times replicate. Data are expressed as the mean ± S.D. n=3, *p < 0.05, **p < 0.01, ***p < 0.001 for Figure 8B-C.

PD-L1 expression on AGS, A549, and MDA-MB-231 cancer cell lines under *ex vivo* organotypic culture with the functional hydrogel was assessed by flow cytometry (**Figure 8A-B**). The expression rate was compared with the tumor cell only group (mock). PD-L1 was strongly expressed on AGS, A549, and MDA-MB-231 cells under non-treated conditions. However, when treated with the functional hydrogel, PD-L1 expression decreased. Meanwhile, PD-L1 mean fluorescent intensity (MFI) on all three cancer cell lines were increased compared to the mock group (**Figure 8C**). Immunofluorescent observation shows that tumor co-culture models showed strong expression of PD-L1 (**Figure 8D and Figure S8**). Notably, the group of MDA-MB-231 demonstrated a markedly higher gene expression compared to both A549 and AGS. These findings are consistent with previous reports. Zheng et al. tested that MDA-MB-231 cells had the highest level of PD-L1 expression among various cancer cells in a co-culture system, and confirmed that this related to their higher immunosuppressive effects 82. In a functional hydrogel containing LECs, we observed differential immune evasion among cancer cell types through co-culture with activated T cells, as indicated by PD-L1 expression. This highlights the potential applicability of our system in elucidating tumor immune evasion mechanisms and evaluating the efficacy of cancer therapies. We plan to conduct *in vitro* and *in vivo* experiments in the further study to evaluate the robust cellular activity of T-LEC and STC.

## Conclusions

This study shows that TMSC-induced LECs (T-LECs) help sustain CD8^+^ T cell activation when exogenous antigens or stimulating cytokines are added within a biomimetic hydrogel under a malignant TME. In summary, the 10% 5:1 GH-GMA exhibited suitable physical properties for cell and cytokine delivery among various combinations of Gel-GMA and HA-GMA. The tonsil was served as a good tissue source to obtain TMSCs that can differentiate into LECs, and CD8^+^ T cells for T cell stimulation. The cellular characteristics of TMSCs and CD8^+^ T cells isolated from the tonsil, as well as LECs differentiated from TMSCs and activated T cells, were confirmed through their respective specific markers or cytokine production. To activate, maintain, or expand T cells *ex vivo*, we used a functional hydrogel with 10% 5:1 GH-GMA loaded anti-CD28, IL-2 and VEGF-C, which encapsulates T-LECs. It was revealed that the activated T cells proliferated and migrated toward T-LEC spheroids in the functional hydrogel within 3 days, as evidenced by staining and the confirmation of T cell migration-related genes. Furthermore, we found that LECs help sustain T cell activation by IL-2 secretion. Finally, activated T cells, T-LEC spheroids, and tumor cells were co-encapsulated in the functional hydrogel to make TME mimic organotypic culture, resulting in a notable increase in chemokines and molecules related to T cell migration and apoptosis, and PD-L1 as immune checkpoint. Particularly, the co-culture with MDA-MB-231 showed a remarkable increase in these factors compared to other tumor models.

We anticipated that these results were from the harmony of T-LECs, exogenous cytokines (IL-2 and VEGF-2), and GH-GMA hydrogels for T cell function. In particular, we anticipate that T-LECs can act as APCs, thereby enhancing the activation of T cells. Additionally, it is expected that the sustained release of IL-2 and VEGF-C encapsulated within the hydrogel would respectively influence the activation of T cells and lymphangiogenesis through LECs. GH-GMA may provide a foundation for immune system modulation and cell delivery, and it is likely to have been utilized as a sustained release vehicle for cytokines in the *ex vivo* 3D organotypic culture. Our functional hydrogel, composed of GH-GMA loaded with IL-2 and VEGF-C encapsulating T-LEC spheroids, holds promise as a fundamental platform for cancer immunotherapy, such as immune checkpoint inhibitors, or for engineering platforms to modulate the suppressive TME.

## Supplementary Material

Supplementary materials and methods, figures and tables.

## Figures and Tables

**Table 1:** Composition of GH-GMA depending on mixture ratio with HA-GMA and Gel-GMA.

Sample	Composition	
	Gel-GMA (g/ml, % of w/v)	HA-GMA (g/ml, % of w/v)
10% Gel-GMA	0.1 (10%)	0 (0%)
5% HA-GMA	0 (0%)	0.05 (5%)
3:1	0.075 (7.5%)	0.025 (2.5%)
10% GH-GMA 5:1	0.0834 (8.34%)	0.0167 (1.67%)
7:1	0.0875 (8.75%)	0.0125 (1.25%

**Table 2:** Gel point and Storage modulus variation based on the content of HA- and Gel-GMA in 10%GH-GMA composites

Parameters	3:1 GH-GMA	5:1 GH-GMA	7:1 GH-GMA	HA-GMA	Gel-GMA
HA-GMA contents (w/v %)	2.5	1.7	1.2	5	0
Gel-GMA contents (w/v %)	7.5	8.3	8.8	0	10
Gel point time (m)	2.2	1.2	0.4	3.5	1.3
Storage modulus G' (Pa) at gel point	71.4	2	0.3	120.1	0.6
Complex viscosity (Pa·s) at gel point	11.5	0.2	0.1	27.0	0.1
Equilibrium storage modulus G' (Pa)	558.9	216.3	12.6	256.1	47.6
Equilibrium loss modulus G'' (Pa)	89.7	4.8	0.7	125.1	0.3
Equilibrium complex viscosity (Pa·s)	90.1	34.4	2.0	45.4	7.6
