# Supplementary material for: Ex vivo enhancement of CD8+ T cell activity using functionalized hydrogel encapsulating tonsil-derived lymphatic endothelial cells
Source: Theranostics. 2025 Jan 1;15(3):850–74. doi: 10.7150/thno.100079 (PMC11700866; doi:10.7150/thno.100079)
Supplement: Supplementary file 1 — Supplementary materials and methods, figures and tables. [file thnov15p0850s1.pdf]

Supplementary data

Ex vivo enhancement of CD8+ T cell activity using functionalized hydrogel encapsulating tonsil-derived lymphatic endothelial cells

Heesun Hong,[1,#] Chan Hum Park,[1,2,#] Ji Seung Lee,[1] Kyunghee Kim,[1] Sudarshini Nath,[1] Moon Sik Oh,[1] Sol Kim,[1] Chul Hee Lee,[1] Ki Hyun Kim,[1] Woo Hee Choi,[3] Kyu Young Choi,[1,4] Hae Sang Park,[1,2] Ok Joo Lee,[1] In-Sun Hong,[5] Soon Hee Kim[1,*]

[1]*Nano-Bio Regenerative Medical Institute, College of Medicine, Hallym University, Chuncheon 24252, Republic of Korea*

[2]*Departments of Otorhinolaryngology-Head and Neck Surgery, Chuncheon Sacred Heart Hospital, School of Medicine, Hallym University, Chuncheon 24252, Republic of Korea.*

[3]*R&D Institute, ORGANOIDSCIENCES Ltd., Seongnam 13488, Republic of Korea*

[4]*Department of Otorhinolaryngology-Head and Neck Surgery, Hallym University College of Medicine, Kangnam Sacred Heart Hospital, Seoul 07441, Republic of Korea*

[5]*Department of Molecular Medicine, School of Medicine, Gachon University, Incheon 21565, Republic of Korea*

[#]Heesun Hong and [#]Chan Hum Park contributed equally to this work.

* Corresponding author: Soon Hee Kim, Nano-Bio Regenerative Medical Institute Hallym University, 1 Hallymdaehak-gil, Chuncheon, Gangwon-do, 24252, Republic of Korea

E-mail: shkim@hallym.ac.kr, Tel.: 82-33-248-2509, Fax: 82-33-251-0503

Materials and Methods

1. Synthesis of modified gelatin and hyaluronic acid with glycidyl methacrylate

5 g of gelatin from porcine skin (90-100 kD, bloom 300, Type A, Sigma-Aldrich, St. Louis, MO, USA) was dissolved in 250 ml of distilled water (DW) at 60 °C for 1 hour then stirred until completely dissolved. Gelatin solution was maintained at 60 °C with stirring at 600 rpm for 30 min, and adjusted pH 3.5 with 1 M HCl. After 10 min, 10 ml of glycidyl methacrylate (mM, GMA, Sigma Aldrich) solution was added into the gelatin solution using syringe (Gel-GMA) with constant stirring at 60 °C. The Gel-GMA solution was stirred in a fume hood with continuous stirring at 60 °C. The following day, Gel-GMA solution was filtered through a miracloth (Millipore, Burlington, MA., USA), and dialyzed using dialysis membrane (MWCO 12–14 kDa, Spectrumlabs, Rancho Dominguez, CA, USA) for 7 days against distilled water (DW) in shaker at 70 rpm maintained at 40 °C. DW was changed three times a day. After 7 days dialysis, Gel-GMA solution was filtered using miracloth, and prepared for mixing with HA-GMA solution for the further study.

For the fabrication of HA-GMA, 1 g of Hyaluronic acid (HA, 15 kDa, Shiseido, Japan) was dissolved in 100 ml of PBS and stirred for 3 h. 7.5 ml of triethylamine (TEA., Sigma-Aldrich) was added, and stirred for 1 hour at RT. 7.5 g of tetrabutyl ammonium bromide (TBAB., Sigma-Aldrich) was added into HA-TEA solution, and allowed to dissolve completely with stirring at 600 rpm. After 1 hour reaction, 7.5 ml of GMA was added into reacted HA solution with TEA and TBAB. TEA and TBAB acts as phase transfer catalyst for enhancing the chemical reaction of aqueous HA and non-aqueous GMA molecules. The HA solution was reacted with stirring at RT with gentle agitation for overnight. The following day reacted HA solution was dialyzed using dialysis membrane (MWCO 6-8 kDA, Spectrumlabs, Rancho Dominguez, CA, USA) for 7 days against distilled water at RT with changing DW 3 time a day. After dialysis, HA-GMA solution was prepared for the mixture with Gel-GMA. GH-GMA was fabricated by physically mixing Gel-GMA and HA-GMA with different compositional ratios (3:1, 5:1, and 7:1).

2. Characterization and measurements of GH-GMA

^{1}H-NMR: To determine the degree of methacrylation, GH-GMA and other control materials were analyzed by ^{1}H-NMR at a frequency of 400 MHz using a Bruker DPX FT-NMR Spectrometer (9.4 T) from Bruker Analytik GmbH (Karlsruhe, Germany). Deuterium oxide (D_2O, Sigma-Aldrich) was used

as the solvent, with 700 μL per 5 mg of sample. All samples were filtered using a 0.45 μm filter before analysis. Baseline correction was applied before measuring the integrals of the peaks of interest using ACD/NMR Processor Academic Edition.

Fourier Transform-Infrared Spectroscopy (FT-IR) analysis. Fourier Transform-Infrared Spectroscopy (FT-IR, frontier FT-IR Spectrometer, PerkinElmer, Waltham, MA, USA) analysis was performed for freeze-dried Gel-GMA, HA-GMA, 3:1, 5:1, and 7:1 GH-GMA. Each sample was placed directly onto the stage of the FT-IR spectrometer instrument, and each spectrum was acquired in absorbance with 32 scans, at 4 cm^{-1} resolution, and 4000 ~ 500 cm^{-1} spectral range.

X-ray diffraction (XRD). The chemical composition and crystallinity of samples were analyzed with X-ray diffraction instrument (XRD, D8 ADVANCE with DAVINCI, Bruker, Karlsruhe, German) with CuKα radiation and LynxEye XE detector. The XRD patterns were recorded at the rate of 0.5 sec/step, 0.02 step, at 40 kV and 40 mA in the range of 2θ = 5–60°.

Rheological Analysis and Characterization of GH-GMA. The gel point of the hydrogel with different concentrations of GH-GMA with Gel-GMA and HA-GMA as control were measured using Rheometer (Anton Paar Physica, Ostfildern, Germany) MCR 501 testing equipment and an Anton Paar CP25-1/TG with 25 mm diameter cone-plate geometry). For the measurements, 200 μl of each hydrogel solution was introduced to the middle portion of a glass plate (CTD600/UV). The test was performed at ambient temperature with a frequency of 1 rad s^{-1}, shear amplitude of 0.1%, and a UV (365 nm) intensity of 3 mW/cm^2 for 500 s.

Scanning Electron Microscopy (SEM). The cross-sectional microscopic observations of freeze-dried Gel-GMA, HA-GMA, 3:1, 5:1, and 7:1 GH-GMA were performed by ultra-high resolution scanning electron microscopy (SEM, Hitachi S-4800, Hitachi, Tokyo, Japan). Each freeze dried samples were fixed using a carbon conductive adhesive tape on aluminum stubs and covered with gold palladium using a sputter coat at 7 mA for 60 s using specimen coater (Leica EM ACE600, Leica, Wetzlar, Germany)

In vitro swelling test. Prepared hydrogels were sized with approximately diameter 10 mm and thickness 3 mm after photopolymerization using UV (365 nm) exposure, and weighed as an initial weight. Then, hydrogels were immersed in PBS at 37 °C shaking incubator at 45 rpm for up to 360 min, and taken from the 6 well plate and weighed as a swelling sample at time points.

$$Swelling\ ratio\ (\%) = (Wet - Dry) / Dry * 100$$

Biodegradation assay. Degradation assay for 10% of 3:1, 5:1, and 7:1 GH-GMA along with 10% Gel-GMA and 5% of HA-GMA as control hydrogel were examined at the time points of 0, 2, 6, 12, 18, 24, 30, 36, and 48 hrs. Each photopolymerized hydrogel were weighed (W0) after UV exposure, and placed into 1 ml eppendorf tube contained PBS with 40 mU/ml hyaluronase, and incubated in shaking incubator at 37 °C with speed at 70 rpm constantly. At designated time points, each different concentration of hydrogels was taken out from the PBS solution and freeze-dried as a dry weight (Wt). The degradation rate (DR) was determined as follows:

$$\% \text{ of } DR = (W0 - Wt) / W0 * 100$$

In vitro cytokine release Assay. Released cytokine contained supernatants were obtained from the 5% HA-GMA, 10% Gel-GMA, 10% of 3:1, 5:1, and 7:1 GH-GMA hydrogel at the time points of 0, 6, 12, 24, 36, 48, 56, 72, 96, 120, 144, and 168 h. Prepared each concentration of hydrogels were sized with approximately diameter 10 mm and thickness 3 mm. Hydrogels were placed into 24 well plate, added serum free RPMI1640 media, and covered with lid to prevent of medium evaporation. Cytokine release experiments were performed in a shaking incubator at 37 °C with constant shaking at 45 rpm. Supernatants were collected into 1 ml Eppendorf tube at each time point, and frozen at -80 °C for ELISA. Released VEGF-C and IL-2 were measured using VEGF-C ELISA assay (human VEGF-C quantikine ELISA kit, R&D Systems) and IL-2 (Abcam, Cambridge, UK) according to manufacturer's instruction. The optical density values were determined at a wavelength of 450 nm using spectrophotometer (BioTek instruments, Winooski, VT., USA) with non- cytokine loaded hydrogel was as a control.

3. Induction & confirmation of *in vitro* trilineage differentiation from TMSCs

Adipogenesis. Cultured T-LECs at passage 3 were plated 1 x 10^4/ml in 48 well plate with DMEM supplemented with 10% FBS and 1% PS for 2 days cultivation. TMSCs culture media were removed, and cells were washed twice with DPBS (Dulbecco's phosphate-buffered saline, calcium-, magnesium-free, Corning, NY., USA). And adipogenesis differentiation Medium (StemPro^{TM} Adipogenesis Differentiation Kit, Gibco, Carlsbad, CA., USA) was added to cells, and cells were cultured for 3 weeks with changing media every 3–4 days. After 3 weeks, adipogenic differentiation ability was detected by OilRed O staining method for assessing lipid accumulation. In details, induced cells were rinsed twice with DPBS, fixed with 4% PFA (paraformaldehyde, Thermo Fisher Scientific, Waltham, MA., USA) for 30 min at RT, rinsed with PBS. Subsequentially, incubated cells with 60%

isopropyl alcohol for 5 min at RT, and then removed the remained isopropyl alcohol and dried up completely. And stained with 2% OilRed O solution (Sigma-Aldrich) for 30 min at room temperature, and removed the remained OilRed O solution and washed the cells 5 times with PBS. Stained cells were observed under inverted microscope (Nikon, Japan).

Chondrogenesis. 2.0×10^7/ml of TMSCs at passage 3 were suspended in chondrogenic differentiation medium (StemPro™ Chondrogenesis Differentiation Kit, Gibco, Carlsbad, CA., USA). 5 µl droplet of cell suspension was seeded in the center of 48 well plate for the generation of micromass culture. After cultivation for 3 h under 5% CO_2 incubator maintained 37 °C, and carefully added warm chondrogenic differentiation medium to culture plates, then incubated in 5% CO_2 incubator maintained 37 °C for 3 weeks with refeeding culture medium every 3-4 days. Cultured cells were washed 3 times with PBS, then fixed with 4% PFA at RT for 30 min. After fixation, rinse well with PBS, and stained cells with 1% Alcian Blue solution (Lifeline Cell Technology, Carlsbad, CA., USA) for 30 min at RT. And then stained cells were washed 3 times with 3% acetic acid, then added PBS. Images were taken on an inverted microscope (Nikon, Japan).

Osteogenesis. 5 x 10^4/ml of cultured TMSCs at passage 3 were seeded in 48-well plates with DMEM supplemented 10% FBS and 1% PS, and cultured for 2 days in 5% CO_2 incubator maintained 37 °C. After 2 days cultivation, removed MSCs culture medium, and washed with PBS (Dulbecco's phosphate-buffered saline, calcium & magnesium-free, Corning, NY., USA). And then, added warmed Complete Osteogenesis Differentiation Medium (StemPro™ Osteogenesis Differentiation Kit, Gibco, Carlsbad, CA., USA), and cells were cultured for 4 weeks with changing every 3–4 days. After 3 weeks, cells were rinsed with DPBS, fixed with 4% PFA for 1 h at RT, rinsed twice with PBS, stained with 2% Alizarin Red (pH4.2, Sigma-Aldrich) for 30 min at RT, and rinsed with PBS. Stained cells were detected with inverted microscope (Nikon, Japan).

Table S1. Gene primer sequences

Abbreviations: OCT4, Octamer-binding Transcription Factor 4; SOX2, SRY-box 2; NANOG, ; Nanog Homeobox; LPL, Lipoprotein lipase; FABP4, Fatty acid binding protein4; PPAR-γ, Peroxisome proliferator-activated receptor gamma; SOX9, SRY-Box Transcription Factor 9; COL2A1, Collagen Type II alpha 1 chain; OC, Osteocalcin; ALP, Alkaline phosphatase; BMP2, Bone Morphogenic protein 2; TMSC-derived LECs, Tonsil Mesenchymal Stem Cells; CD31, Cluster of Differentiation 31; KDR, Kinase insert domain receptor; VEGFR3, Vascular endothelial growth factor receptor 3; Prox 1, Prospero homeobox1; LYVE1, Lymphatic vessel endothelial hyaluronan receptor 1; CXCR, C-X-C chemokine Receptor; CXCL, C-X-C chemokine ligand; CCL, C-C Motif Chemokine Ligand; CCR, C-C Motif Chemokine Receptor; ICAM-1, Intracellular Adhesion Molecule-1; LFA-1, Lymphocyte Function-associated Antigen-1; VCAM-1, Vascular Cell Adhesion Molecule-1; MMP, Matrix Metalloproteinase; PRF1, Perforin 1; GZMB, Granzyme B; GAPDH, Glyceraldehyde 3-phosphate dehydrogenase

Applications	Gene	Sequence (5'-3')		NCBI accession
Stemness	Oct-04	Forward:	CCTTCGCAAGCCCTCATTTC	NM_002701.6
		Reverse:	TAGCCAGGTCCGAGGATCAA	
	SOX2	Forward:	CATGAAGGAGCACCCGGATT	NM_003106.4
		Reverse:	TAACTGTCCATGCGCTGGTT	
	NANOG	Forward:	AATGGTGTGACGCAGGGATG	NM_001297698.2
		Reverse:	TGCACCAGGTCTGAGTGTTC	
	c-Myc	Forward:	CACCAGCAGCGACTCTGA	NM_002467.6
		Reverse:	GATCCAGACTCTGACCTTTTGC	
Adipogenesis	LPL	Forward:	CCGCCGACCAAAGAAGAGAT	NM_000237.3
		Reverse:	TAGCCACGGACTCTGCTACT	
	PPAR-γ	Forward:	TCGAGGACACCGGAGAGG	NM_138712.5
		Reverse:	CACGGAGCTGATCCCAAAGT	
	FABP4	Forward:	ATGGGGGTGTCCTGGTACAT	NM_001442.3
		Reverse:	ACGTCCCTTGGCTTATGCTC	
Chondrogenesis	COL2A1	Forward:	TGCATGAGGGCGCGGTA	NM_033150.3
		Reverse:	GGTCCTGGTTGCCGGACAT	
	Aggrecan	Forward:	CTTCCGCTGGTCAGATGGAC	NM_001135.4
		Reverse:	CGTTTGTAGGTGGTGGCTGT	
	SOX9	Forward:	GAAGGACCACCCGGATTACA	NM_000346.4
		Reverse:	GCCTTGAAGATGGCGTTGG	
Osteogenesis	OC	Forward:	TCACACTCCTCGCCCTATTG	NM_199173.6
		Reverse:	GGGTCTCTTCACTACCTCGC	
	ALPL	Forward:	TCCGCGCCCGCTATCC	NM_000478.6
		Reverse:	CAATCGACGTGGGTGGGAGG	
	BMP2	Forward:	ACTCGAAATTCCCCGTGACC	NM_001200.4
		Reverse:	CCACTTCCACCACGAATCCA	

TLECs	CD31	Forward:	GGTCAAAGGATCAGACGACCT	NM_000442.5
		Reverse:	GGTGGTGGCAAGGGACTAAG	
	KDR	Forward:	CGGTCAACAAAGTCGGGAGA	NM_002253.4
		Reverse:	CAGTGCACCACAAAGACACG	
	VEGFR3	Forward:	GACTGTGGCTCTGCCTGG	NM_182925.5
		Reverse:	GGTGTCGATGACGTGTGACT	
	Prox1	Forward:	GAGCCTCCCATTACTCAGACC	NM_002763.5
		Reverse:	GGAGGCTCCCGCTTAGAAAC	
	LYVE1	Forward:	CACACCTGAGGCTGGTTTCT	NM_006691.4
		Reverse:	GCAGTCCTGAGCTGATTCCA	
	PDPN	Forward:	CAACAGTGACCCTGGTTGGA	NM_006474.5
		Reverse:	GCGAGTACCTTCCCGACATT	
Chemokines	CXCR3	Forward:	TTCGCCCACAGTCTGACTTC	NM_001504.2
		Reverse:	AATGGTGCAGTCCTCAGAGC	
	CXCR5	Forward:	GAAGAGTAGGCTGGCCTGTG	NM_001716.5
		Reverse:	TGACTCTGTGTTTCCTGCCC	
	CCR7	Forward:	CACAGCCTTCCTGTGTGGTTT	NM_001838.4
		Reverse:	ACCAGCACGCTTTTCATTGG	
	CCL19	Forward:	CTCACACATTCACCGTTGGC	NM_006274.3
		Reverse:	TGGGGAAGTCCAGAGAACCA	
	CCL21	Forward:	AGGCAGAGCTATGTGCAGAC	NM_002989.4
		Reverse:	CCTTTAGGGGTCTGTGACCG	
Adhesion molecules	ICAM-1	Forward:	TCTTCCTCGGCCTTCCCATA	NM_000201.3
		Reverse:	AGGTACCATGGCCCCAAATG	
	LFA-1	Forward:	CTTGGAGACACATCCCCAAAGA	NM_001127491.3
		Reverse:	CTCGGAGTCCACTTGCTGTC	
Extracellular matrix proteins	MMP8	Forward:	ATGTGACGGGGAAGCCAAAT	NM_001304442.2
		Reverse:	AAAACCACCACTGTCAGGCA	
	MMP9	Forward:	GGTGATTGACGACGCCTTTG	NM_004994.3
		Reverse:	CTGTACACGCGAGTGAAGGT	
Endogenous cytotoxic proteins	PRF1	Forward:	CTATACGGGATTCCAGCTCCA	NM_005041.6
		Reverse:	ACCTTTGTGTGTCCACTGGG	
	GZMB	Forward:	GATCATCGGGGGACATGAGG	NM_004131.6
		Reverse:	GGTCGGCTCCTGTTCTTTGA	
Internal Control	GAPDH	Forward:	CGACCACTTTGTCAAGCTCA	NM_002046.7
		Reverse:	AGGGGTCTACATGGCAACTG	

Table S2. Components and description of Kit for flow cytometry

Vial	Contents	Purpose
hMSC Positive Cocktail	CD90 FITC (Clone: 5E10)<br>CD105 PerCP-Cy5.5 (Clone: 266)<br>CD73 APC (Clone: AD2)	Cocktail to positively identify hMSCs
hMSC Positive Isotype Control Cocktail	mIgG1, κ FITC (Clone: X40)<br>mIgG1, κ PerCP-Cy5.5 (Clone: X40)<br>mIgG1, κ APC (Clone: X40)	Corresponding Isotype Control for hMSC Positive Cocktail
PE hMSC Negative Cocktail	CD34 PE (Clone:581)<br>CD11b PE (Clone: ICRF44)<br>CD19 PE (Clone: HIB19)<br>CD45 PE (Clone: HI30)<br>HLA-DR PE (Clone: G46-6)	Cocktail to identify potential contaminants
PE hMSC Negative Isotype Control Cocktail	mIgG1, κ PE (Clone: X40)<br>mIgG2a, κ PE (Clone:G155-178)	Corresponding isotype control for PE hMSC Negative Cocktail
FITC Mouse Anti-human CD90	CD90 FITC (Clone: 5E10)	Compensation control
PE Mouse Anti-Human CD44	CD44 PE (Clone: G44-26)	Compensation control/MSC positive drop-in
PerCP-Cy™5.5 Mouse Anti-Human CD105	CD105 PerCP-Cy™5.5 (Clone: 266)	Compensation control
APC Mouse Anti-Human CD73	CD73 APC (Clone:AD2)	Compensation control
PE Mouse IgG2b, κ Isotype Control	mIgG2b, κ (Clone: 27-35)	Corresponding Isotype Control for PE Mouse Anti-Human CD44, when used as a drop in

*BD Biosciences, BD Stemflow™, Human MSC Analysis Kit, 562245

Table S3. Other antibodies for flow cytometry

Abbreviations: APC, Allophycocyanin; PE, Phycoerythrin; FITC, Fluorescein isothiocyanate; VEGFR3, Vascular Endothelial Growth Factor Receptor 3; LYVE-1, Lymphatic Vessel Endothelial Hyaluronan Receptor 1; Prox1, Prospero Homeobox 1: CD, Cluster of Differentiation; PD-L1, Programmed cell death protein-Ligand 1; IFN-γ, Interferon-gamma; IL2, Interleukin2.

Application	Antibody Name	Fluorophore	Company
Flow Cytometer	Prox-1	Alexa Fluore®488	Bioss
	LYVE-1	Alexa Fluore®488	R&D System
	CD69	PE	Miltenyi Biotec.
	Podoplanin	APC	Biolegend
	VEGF-R3 PE	PE	
	CD4	PE	
	CD8	APC	
	CD33	FITC	
	CD45RA	FITC	
	CD62L	PE	
	IL2	PE	
	IFN-γ	FITC	
	PD-L1	PE	

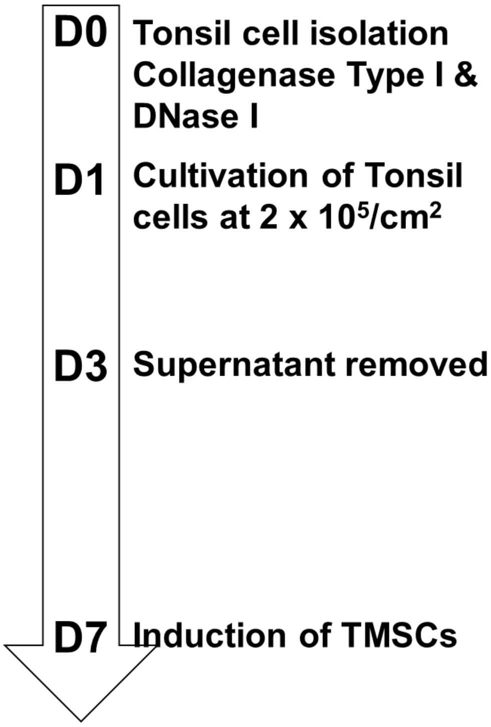

Figure S1. Production process of human tonsil-derived mesenchymal stem cells (hTMSCs).

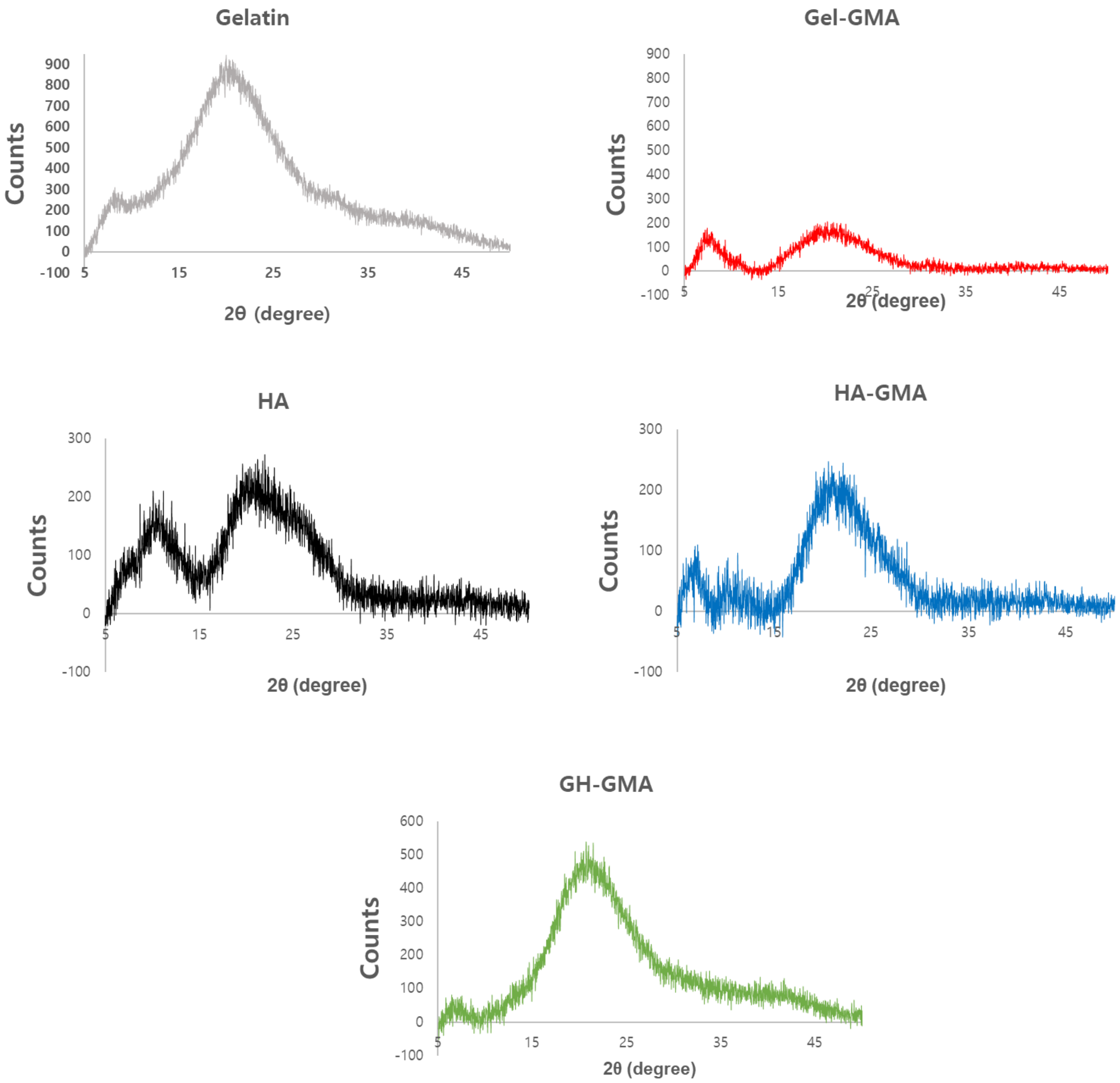

Figure S2. X-ray diffraction (XRD) patterns of each pre-hydrogel

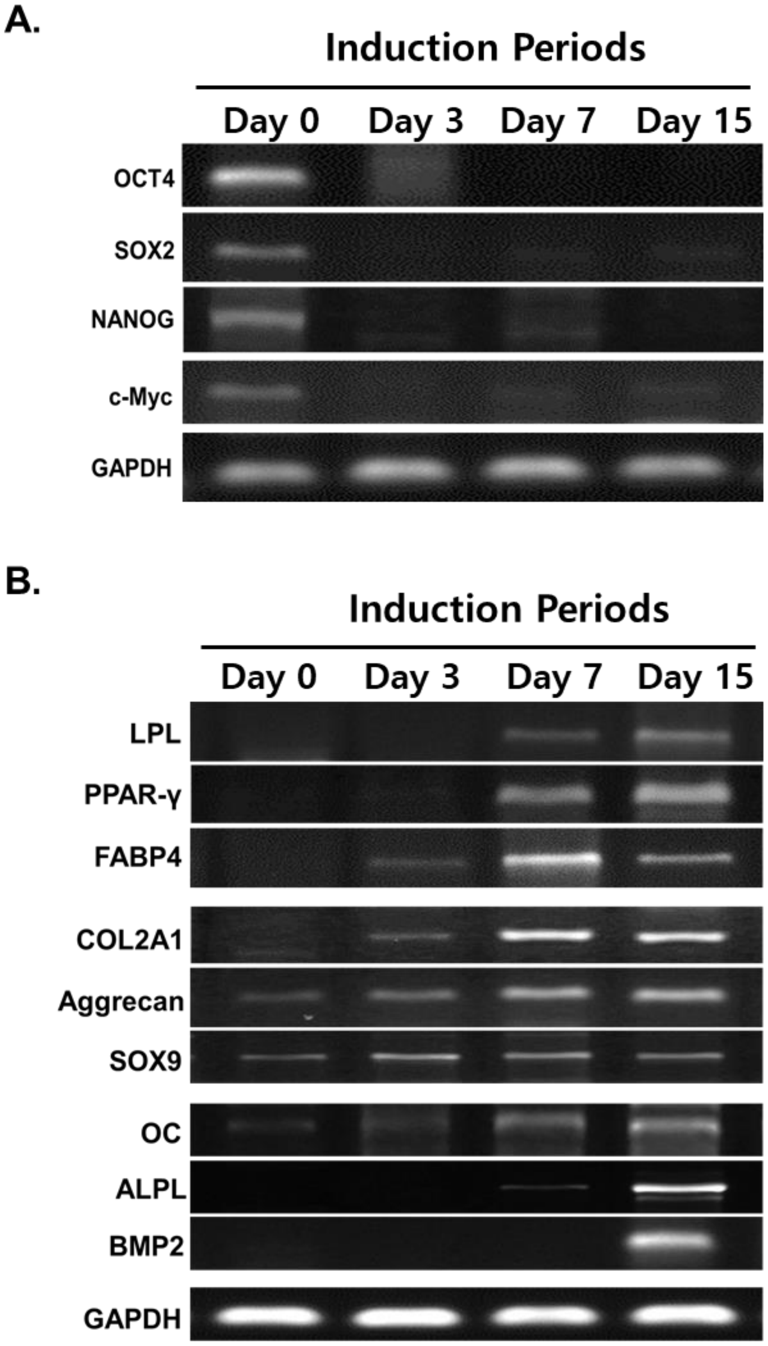

Figure S3. TMSCs characterization by RT-PCR (gel electrophoresis) (A) Stemness confirmation (OCT4, SOX2, NANOG, and c-Myc) of induced TMSCs from passage dependent. Non-induced TMSCs were used as a control. **(B)** Specific gene expression of tri-lineage differentiation from induced TMSCs.

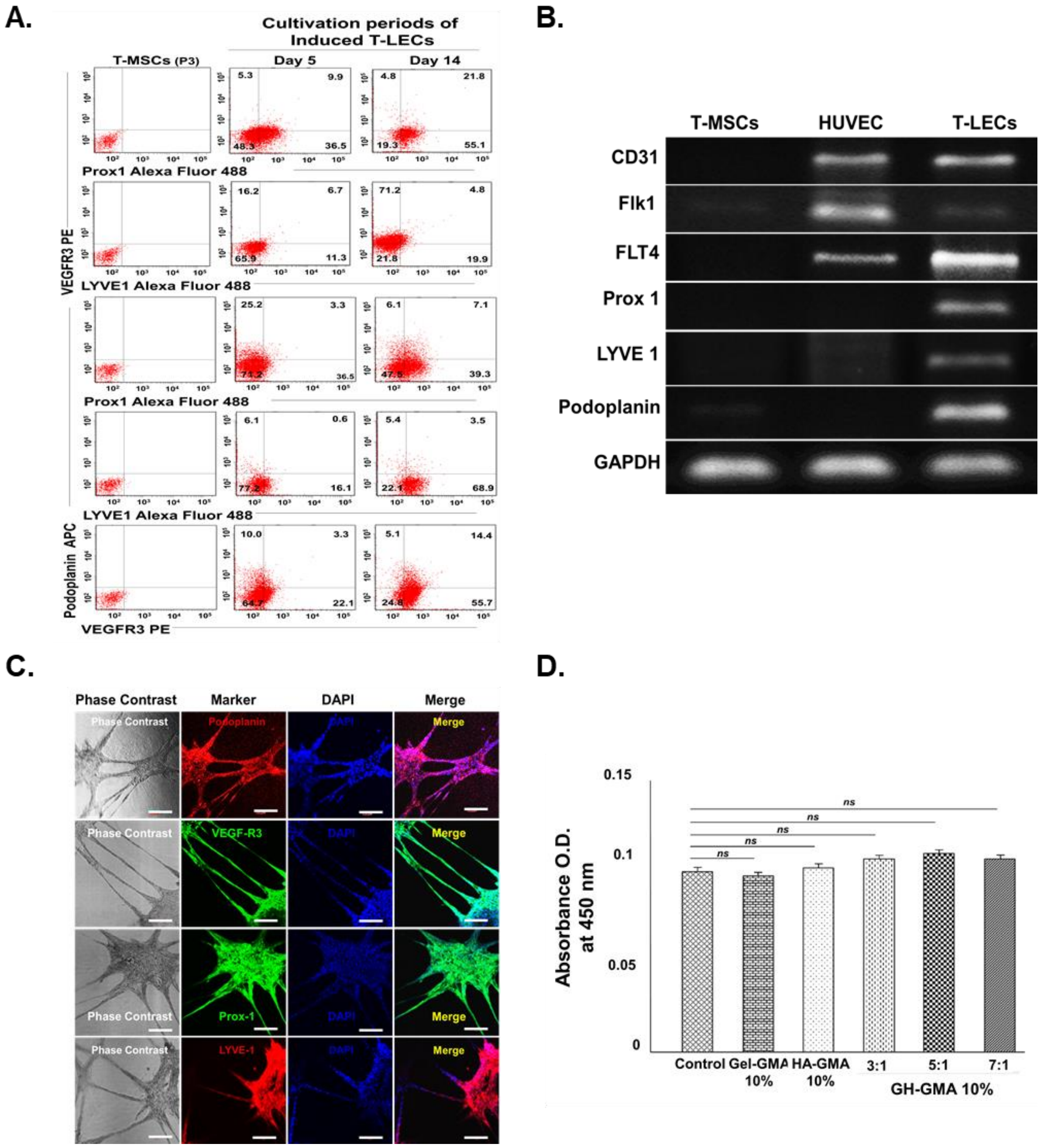

Figure S4. Evaluations of Tonsil derived lymphatic endothelial cells (T-LEC) and T-LEC based spheroids (A) Flow cytometry plots. **(B)** LECs specific gene expression of induced T-LECs by gel electrophosesis (T-LEC: P5). **(C)** Enlarged areas of the small boxes that is highlighted in Figure 4D. Scale bar 100 μm. **(D)** Cell proliferation using CCK 8 assay with T-LEC spheroid encapsulated in 10% Gel-GMA, 5% HA-GMA, and three 10% GH-GMA composite-hydrogels for 72 h. Cell proliferation of T-LEC spheroid was shown no-significant increasing following hydrogel combination. Control (ctrl) was 2D liquid culture media. Data are expressed as the mean ± S.D. *$p < 0.05$, **$p < 0.01$, ***$p < 0.001$. ns = no significant. Experiments were performed three times replicate.

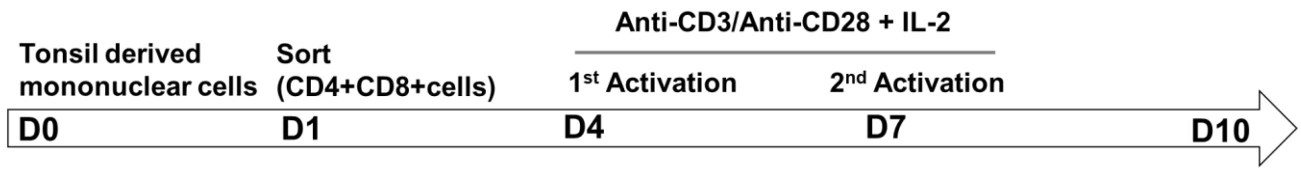

Figure S5. Preparation of stimulated T-CD8+ T cells. The preparation process for the activation and expansion of sorted tonsil-derived CD4+ and CD8+ T cells by anti-CD3 and anti-CD28 antibodies, supplemented with IL-2 over a cultivation period of up to 10 days.

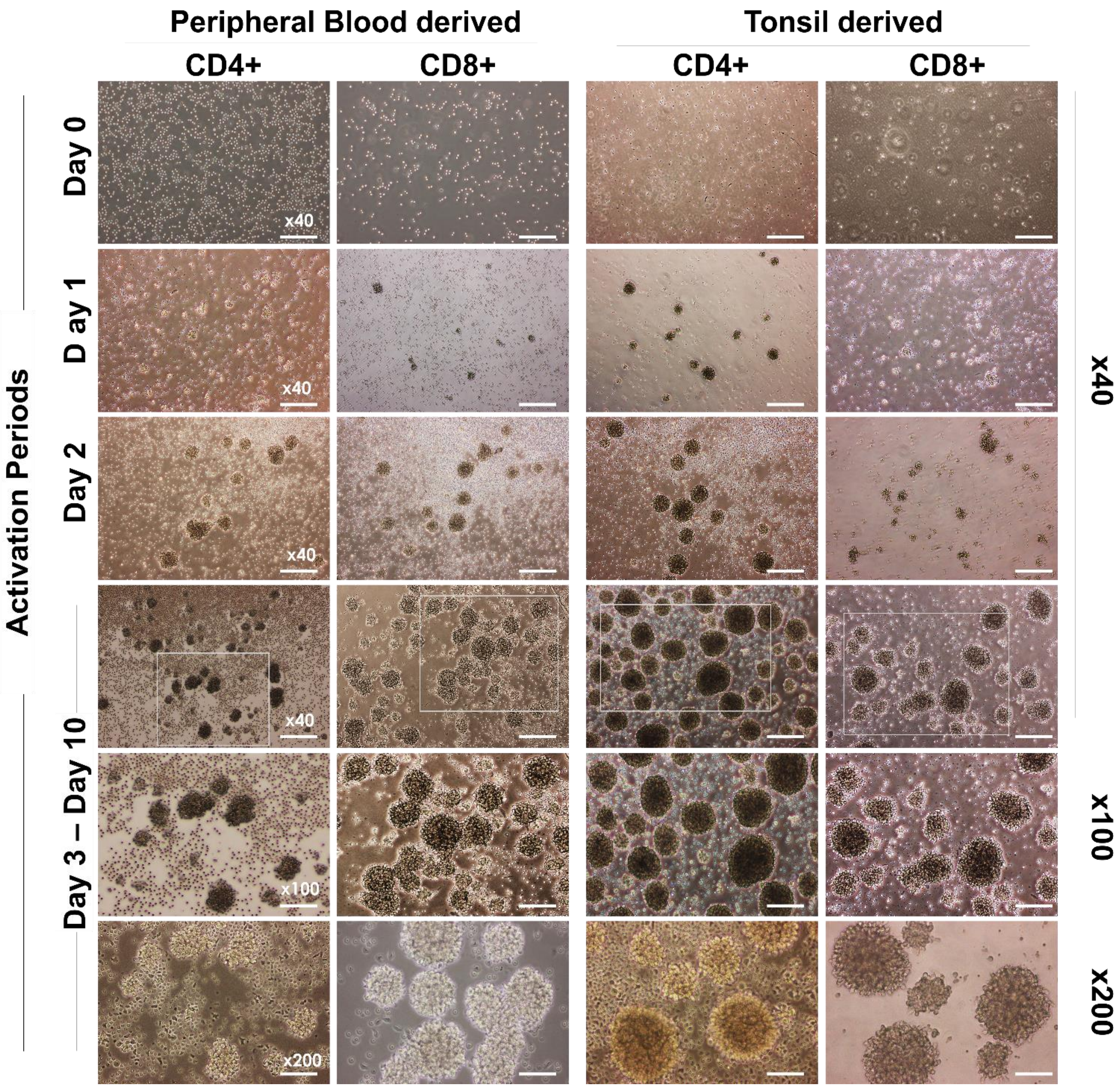

Figure S6. Morphologies during activation and expansion of sorted tonsil derived CD4+ & CD8+ T cells with anti-CD3/anti-CD8 antibodies up to 10 days cultivation. Scale bars= 500 µm (x40), 200 µm (x100), and 100 µm (x200)

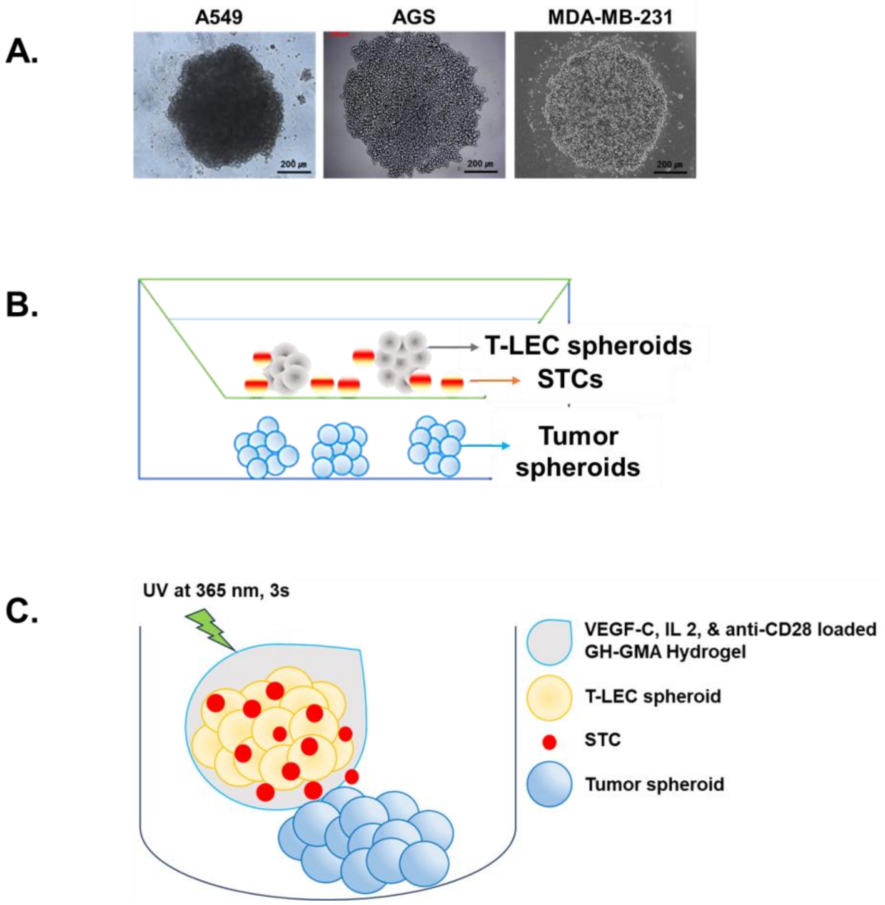

Figure S7. *Ex vivo* reconstituted 3D organotypic culture with cancer spheroids (A) Morphological observation of A549 (lung cancer), AGS (gastric cancer), MDA-MB-231 (TNBC) spheroids. Each cancer cell lines were cultured in low-attached well plate for 7 days. Scale bar = 200 μm. **(B)** STCs migration in transwell assay. The lower chamber of the transwell was seeded with each tumor spheroid and TMSCs as a control. **(C)** A scheme of e*x vivo* reconstituted 3D organotypic culture system with cancer spheroids

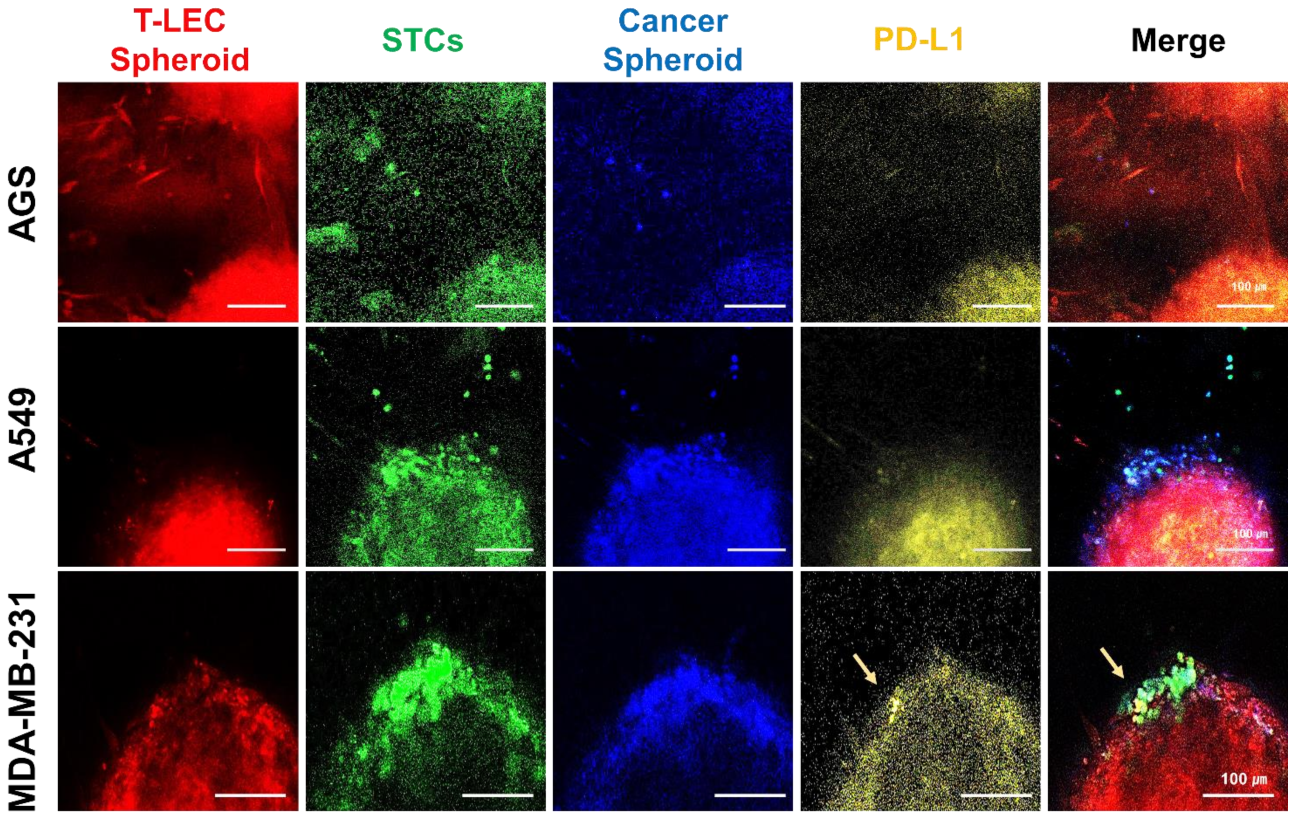

Figure S8. PD-L1 expression from cancer cell lines under *ex vivo* organotypic culture (magnified images from Figure 8D). Immunofluorescent staining of PD-L1 expressed on three cancer cell lines under ex vivo organotypic culture with functional hydrogel containing T-LEC and STCs. Scale bar = 100 µm.
